# How to Design a Relevant Corpus for Sleepiness Detection Through Voice?

**DOI:** 10.3389/fdgth.2021.686068

**Published:** 2021-09-22

**Authors:** Vincent P. Martin, Jean-Luc Rouas, Jean-Arthur Micoulaud-Franchi, Pierre Philip, Jarek Krajewski

**Affiliations:** ^1^Laboratoire Bordelais de Recherche en Informatique, University of Bordeaux, CNRS–UMR 5800, Bordeaux INP, Talence, France; ^2^Sommeil, Addiction et Neuropsychiatrie, University of Bordeaux, CNRS–USR 3413, CHU Pellegrin, Bordeaux, France; ^3^Engineering Psychology, Rhenish University of Applied Science, Cologne, Germany

**Keywords:** sleepiness, speech processing, corpus design, methodological issue, guidelines

## Abstract

This article presents research on the detection of pathologies affecting speech through automatic analysis. Voice processing has indeed been used for evaluating several diseases such as Parkinson, Alzheimer, or depression. If some studies present results that seem sufficient for clinical applications, this is not the case for the detection of sleepiness. Even two international challenges and the recent advent of deep learning techniques have still not managed to change this situation. This article explores the hypothesis that the observed average performances of automatic processing find their cause in the design of the corpora. To this aim, we first discuss and refine the concept of *sleepiness* related to the ground-truth labels. Second, we present an in-depth study of four corpora, bringing to light the methodological choices that have been made and the underlying biases they may have induced. Finally, in light of this information, we propose guidelines for the design of new corpora.

## Introduction

Speaking is a complex task involving multiple muscular, neurological, and cognitive processes. In Krajewski et al. (1), a 2-block model of speech production is proposed. The first block describes the cognitive planning of the tasks, which involves the intention to speak and the creation of the idea, the linguistic programming, and the generation of articulatory targets and neuromuscular commands. The second block concerns the motorical actions, involving respiration, phonation, and articulation. Proprioceptive feedback adjusts the neuromuscular commands based on the motorical actions, whereas auditory feedback corrects it based on the resulting audio speech waves. These two blocks can be completed, depending on the speaking task, by memory and other cognitive actions for spontaneous speech or reading-related cognition when reading texts out loud.

Numerous diseases impact these processes and, thus, can be detected through voice (2). We can differentiate two axes. On one side, the estimation of neurodegenerative pathologies that impact directly and typically the voice production of the subjects, such as pre-symptomatic Hungtington's disease [87% of accuracy reported in Rusz et al. (3)], Alzheimer's disease [80% of accuracy in Weiner et al. (4)], dysphonia [89% of accuracy in Tulics et al. (5)], or Parkinson's disease [84% of accuracy in Vasquez-Correa et al. (6)].

On the other side, the estimation of psychiatric pathologies that impact both motorical actions and cognitive planning, with complex phenomenology. They include bipolar disorders [57.4% of accuracy in Ringeval et al. (7)], autism spectrum [69.4% on four categories in Asgari et al. (8)] or, the most studied so far, depression [88% of accuracy in in Vázquez-Romero and Gallardo-Antolín (9)], a complete review is proposed in Cummins et al. (10).

Among all the other existing psychiatric disorders, we focus in this study on chronic and instantaneous sleepiness. Regarding sleepiness detection through voice, two main objectives are studied in the literature.

- **Chronic sleepiness**. Patients affected by sleep disorders require a customized and regular follow-up. Regrettably, sleep medicine is a domain suffering from too long queuing, resulting in spaced and uneven interviews. Given this situation, a virtual physician has been designed to follow-up patients at home (11, 12), allowing a regular collection of data and the measure of the variation of symptoms in response to the treatment. To integrate chronic sleepiness detection through voice systems into these virtual assistants, two corpora have been designed at the Sleep Clinic of Bordeaux Hospital.
  First, the Maintenance of Wakefulness Test Corpus (MWTc) has been elaborated between 2018 and 2019. Based on the recordings of 75 patients undertaking a Maintenance of Wakefulness Test (MWT) (as shown in section 1.6.2), this corpus suffers from multiple methodology defects that have prevented us from exploiting it. As a consequence, no study has been published based on this corpus. Nevertheless, it has paved the way to other corpora such as the Multiple Sleep Latency Test corpus (termed hereafter MSLTc) and shows interest in being analyzed to discuss these methodological flaws. It is extensively presented in section 3.3.
  The only available studies aiming at estimating chronic sleepiness through voice are based on the MSLTc (13), which contains recordings of 106 patients affected by sleep pathologies. These patients present symptoms such as hypersomnolence, a chronic sleepiness impacting their everyday life. The available labels are the sleep latencies to a medical test measuring their daytime propensity to fall asleep [the Multiple Sleep Latency Test (MSLT)], and the answer to a short-term subjective sleepiness questionnaire [Karolinska Sleepiness Scale (KSS)]. This corpus is extensively presented in section 3.4.
  At this time, the studies based on this corpus only led to preliminary results. In Martin et al. (14), an Unweighted Average Recall (mean of the recall on the two classes—termed hereafter UAR) of 60% has been obtained on the detection of daytime propensity to sleep based on voice biomarkers. The same studies achieved a UAR of 82.6% with a system based on reading errors (15).
  However, the implementation of these systems in medical applications is not ready yet: the first suffers from too low accuracy, and the second was a proof of concept based on manual annotation of the recordings by human annotators, not a fully automated process. These preliminary studies have nevertheless paved the way to other paradigms and applications on objective sleepiness and pathological populations.
- **Immediate sleepiness**. On the other side, most of the studies found in the literature focus on the detection and the estimation of short-term subjective sleepiness on healthy subjects, finding applications in the monitoring of performances for tasks requiring high cognitive loads, such as driving (16). This task has been the subject of two international challenges: the Interspeech 2011 (IS11) challenge on speakers state estimation (17), introducing the Sleepy Language Corpus (SLC), and the Interspeech 2019 (IS19) challenge on continuous sleepiness estimation (18) that introduced the SLEEP corpus (also referred to as the Dusseldorf Sleepy Language Corpus). Based on different corpora, the two challenges share the same way of labeling instantaneous sleepiness with the KSS, a medical questionnaire aiming at measuring instantaneous subjective sleepiness (see section 2.1.1 for more information).
  During the IS11 challenge, the objective was to achieve the highest UAR on binary classification Sleep (SL) and Non-Sleepy (NSL). SL and NSL were delimited by a 7.5 threshold in the KSS. On the six systems that have been proposed for the IS11 challenge, only three outperformed the baseline UAR of 70.3%. The best performances have been obtained by a system based on the ASIMPLS algorithm (19, 20) and reach 71.7%. More recently, a work focusing on the longer reading tasks of the SLC has reported an accuracy of 76.4% (21). Even if this study shows a significant improvement on a subset of the SLC, this performance is still below the necessary 80–85% for medical uses.
  Eight years later, the SLEEP corpus was introduced during the IS19 challenge. This new challenge has brought both a new paradigm and a new corpus: the SLEEP corpus has been introduced for the changing from binary classification (SL vs. NSL) to regression between the estimated label and the ground-truth KSS. Indeed, the SLEEP corpus, with more than 16,000 samples, seemed more suited for regression (that requires more data) than the SLC, which contains 9,000 samples.
  The baseline for this challenge was Spearman's correlation between estimated KSS and the ground truth value of ρ = 0.347. Over the dozen systems that have been proposed, two studies (22, 23) introduced deep learning systems for the first time in the task of sleepiness detection through voice. They achieved respective correlations of ρ = 0.369 and ρ = 0.343 while the winner of the challenge, who used Fischer vectors and bag-of-features (24), achieved a correlation of ρ = 0.387. Since then, two other systems using deep learning have been proposed (25, 26), both achieving performances below the winner of the challenge (resp. ρ = 0.317 and ρ = 0.367).

However, although all these efforts, there still exist pitfalls and problems in analyzing sleepiness through voice. First, regarding sleepiness-related problems, semantic plays an important role. For instance, a recent study has already shown that the wording of questions and the used scales significantly affect the response of the patients when self-assessing their sleep duration (27). In the same manner, the precision of the definition of task impacts the experimental conditions and the labeling of data. If in common language, *lack of vigilance, a drop of performances, fatigue, sleepiness* or *drowsiness* are interchangeable, these terms have different medical definitions, remediation, measures, and expressions through voice. The misunderstanding of such concepts could lead to inappropriate labeling of data, leading to a loss of the meaning of the machine learning algorithm designed on such corpora.

Furthermore, facing the unexploitable performances and the inability for deep learning to produce exploitable systems—although it has brought significant improvements in other fields (28)—a recent study (29) questioned the feasibility of the task: is it even possible to estimate sleepiness through voice? Are the vocal changes induced by sleepiness visible enough to be detected and used for classification? To answer this question, a sub-corpus of 99 samples of the SLEEP corpora has been annotated by 25 trained annotators. The labels obtained in this way have reached a correlation of ρ = 0.72 with the ground-truth KSS and binarizing the KSS label using the same 7.5 threshold as in the SLC, they achieved an UAR of 93.6%. As a consequence, human audition can detect subjective sleepiness through voice. This result implies that the task is feasible and that the unusable performances of the state-of-the-art machine learning algorithms are not a consequence of the difficulty of the task, but the consequence of another issue. Facing this observation, the authors of this study have examined the SLEEP corpus and concluded that there are too many samples per speaker and that they have been recorded in a too restrained diversity of sleepiness states. This creates an intrinsic link between the identity of speakers and their KSS levels, that prevents algorithms from learning the impact of sleepiness through voice. We hypothesize that other biases could exist in corpora, maintaining the glass ceiling of sleepiness through voice performances.

The objective of this study is fourfold. First, we propose a practical definition of sleepiness and related concepts. Second, we describe precisely, based on the previous definitions, the tools used in the corpora to measure sleepiness. Then, we study extensively the four previously mentioned corpora to highlight their strengths and weaknesses and identify their sources. Finally, we propose guidelines to design relevant corpora for sleepiness detection through voice, based on the previous observations.

## 1. Task statement

**Figure 1:**
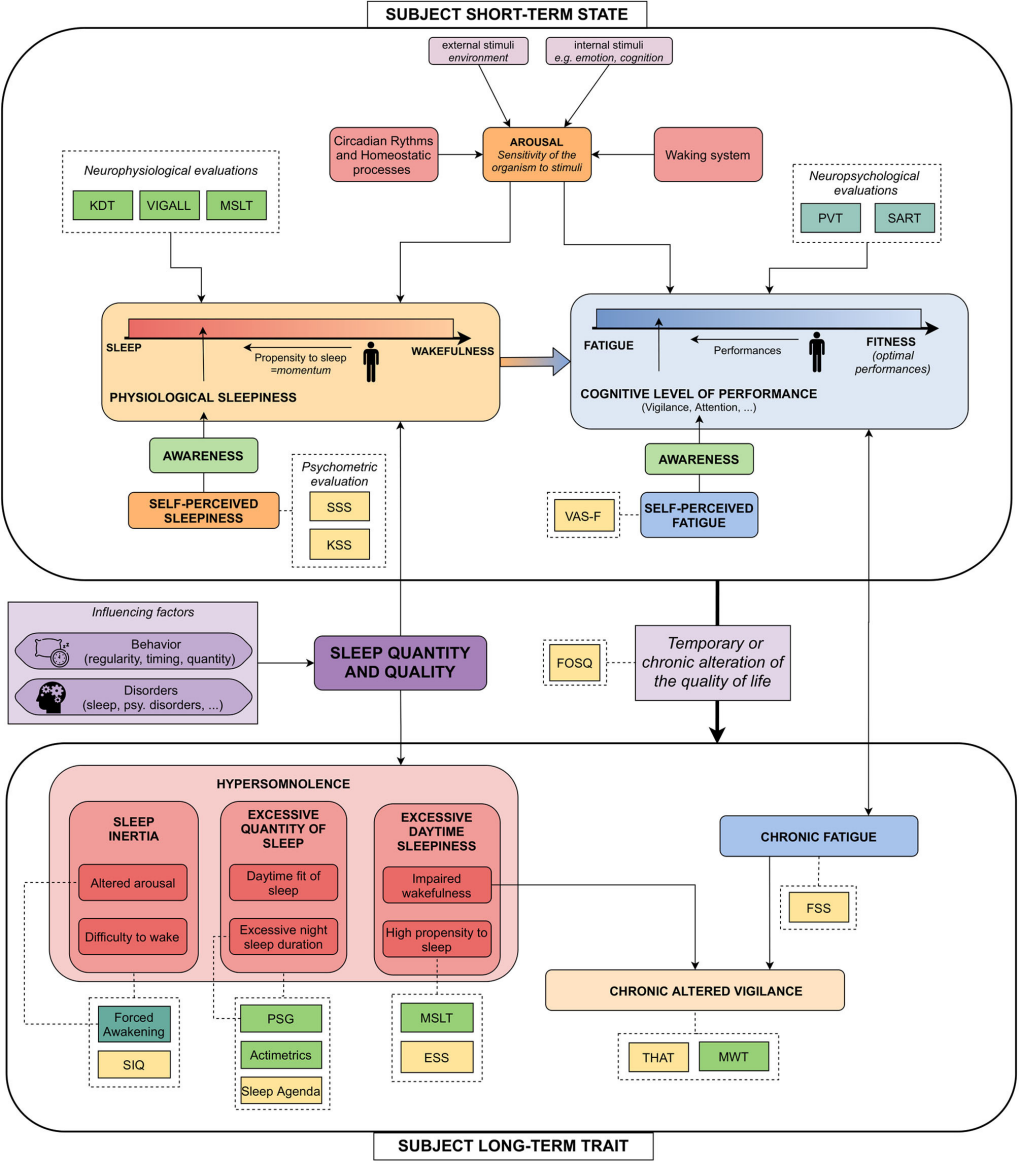
Explicating figure highlighting the links between the commonly blended notions around sleepiness, and their measures. Light green dashed boxes: neurophysiological measures; Forest green dashed boxes: neuropsychological measures; Yellow dashed boxes: psychometric questionnaires. KDT, Karolinska Drowsiness Scale (32); VIGALL, Vigilance Algorithm Leipzig (33); MSLT, Multiple Sleep Latency Test (34); PVT, Psychomotor Vigilance Task (35); SART, Sustained Attention to Response Task (36); SSS, Stanford Sleepiness Scale (37), KSS: Karolinska Sleepiness Scale (32); VAS-F, Visual Analog Scale–Fatigue (38); SIQ, Sleep Inertia Questionnaire (39); Forced Awakening, Event-related potentials during forced awakening (40); PSG, Polysomnography; ESS, Epworth Sleepiness Scale (41); MWT, Maintenance of Wakefulness Test (42); THAT, Toronto Hospital Alertness Test (43); FSS, Fatigue Severity Scale (44); FOSQ, Functional Outcomes of Sleep Questionnaire (45).

Sleepiness is a concept used in multiple studies, but no consensus exists on what exactly this refers to. If some studies restrict this term to the subjective feeling of the patient, it is often mixed up with vigilance or cognitive performance. A better understanding of these concepts would allow a better choice of sleepiness label when designing corpora and give a better insight into the impact of the measured phenomena on voice accordingly. We rely on two recent studies identifying different tools to measure and characterize hypersomnolence (30, 31) and to propose the following practical definitions. The objective of this part is not to give a definitive model but to sensibilize database designers to the multiplicity of sleepiness and its measure. All these phenomena and their measures used in the studied corpora are represented in Figure 1.

### 1.1. Speaker State

To begin with, we introduce notions related to the state of the speaker, i.e., non-chronic phenomena manifesting on durations about minutes or hours (top of the Figure).

- *Arousal* could be defined as the sensitivity of the organism to stimuli (46). It varies with circadian and homeostatic processes, external and internal stimuli, and the waking process. It influences both physiological sleepiness and cognitive performance.
- *Physiological sleepiness* is defined as the state of the subject on the continuum between sleep and wakefulness (30). A related concept is the propensity to sleep, which could be defined as the momentum to go to the lower part of this continuum. Physiological sleepiness is influenced by the level of arousal, but also by the sleep quantity and quality of the subject. It is usually evaluated by neurophysiological measures (KDT, Vigall, or MSLT).
- *Self-perceived sleepiness*—or *subjective sleepiness*—is the awareness one has of physiological sleepiness. It is measured by psychometric scales [Stanford Sleepiness Scale (SSS), KSS].
- *Fatigue* is defined as a “progressive exhaustion of cognitive and physical capacities linked with an effort” (31). It is opposed to *fitness*, i.e., optimal performances. The state of the subject on the continuum going from fatigue to fitness corresponds to its *cognitive level of performance*. To simplify, we grouped *cognitive performances, attention*, and *vigilance* under the same concept, sometimes referred to as *behavioral sleepiness* (30). This concept is usually estimated by neuropsychological measures such as Psychomotor Vigilance Task (PVT), Sustained Attention to Response Task (SART).
- *Self-perceived fatigue*, for its part, is the awareness one has of their cognitive performance and is usually measured by the Visual Analog Scale-Fatigue (VAS-F).

### 1.2. Speaker Trait–Hypersomnolence

Feeling sleepy is a consequence of the natural course of the interaction between the homeostatic balance and the circadian cycle across the day. However, when sleepiness induces a temporary or chronic alteration of the quality of life, it becomes a clinical symptom known as hypersomnolence.

If so, sleepiness loses its instantaneous dimension and becomes a trait of the subject, affecting it for a long period of time (weeks or months). Hypersomnolence can be divided into three main categories, each one subdivided into two subcategories. These are described in Figure 1, in which we also provide numerous sleepiness-related measures. In the following, we will focus on the two measures given by the studied corpora.

First, high propensity to sleep, which is a subcategory of Excessive Daytime Sleepiness (EDS) and studied in the MSLTc. Second, alteration of the wakefulness implies altered vigilance, which is measured by the MWT. As they measure phenomena linked to hypersomnolence—that is a trait symptom of the subjects—objectively, these measures are sometimes referred to as “objective long-term sleepiness,” to differentiate from subjective long-term sleepiness measures and short-term states measures.

## 2. Sleepiness measures

This part introduces the different sleepiness measures that will be encountered later on.

### 2.1. Subjective Sleepiness and Hypersomnolence Measures

The following questionnaires aim at estimating the subjective experience of the subject, being its sleepiness or its hypersomnolence.

#### 2.1.1. Karolinska Sleepiness Scale

The KSS is a medical subjective questionnaire aiming at estimating sleepiness (32). Two versions of it exist: a 1–9 version ranging from “1–Extremely alert” to “9–Very sleepy, great efforts to keep alert, fighting sleep,” and a 1–10 version adding a supplementary item labeled as “10–extremely sleepy, cannot stay awake.” However, these two versions of the questionnaire are medically similar and can be used interchangeably (47). Not requiring much time to fill for the validity of a dozen minutes, the KSS is a reliable and easy way to measure instantaneous sleepiness (32, 48).

The most common approach found in the literature to estimate sleepiness from voice is to simplify the problem into binary classification. To do so, a threshold has to be set to define the SL and NSL classes. Based on the experiment conducted in Krajewski et al. (16), the reference threshold proposed during the IS11 challenge is set to 7.5, the threshold above which no micro-sleep event has been observed during the vocal tasks that were monitored by electroencephalography (EEG). Moreover, an in-depth study about the KSS (48) has concluded that a score higher than 7 is a relevant measure of altered awakening state and cognitive performances, i.e., altered vigilance, comforting this choice.

#### 2.1.2. Epworth Sleepiness Scale

The Epworth Sleepiness Scale (ESS), is an 8 items questionnaire, each one scoring from 0 to 3 the chance to doze in different situations, the resulting score being between 0 and 24 (41). This questionnaire has been elaborated to detect subjective propensity to sleep. It is widely used, for example, to assess chances to doze at the wheel (49, 50), or to assess the need for objective measures such as an MSLT to make a diagnostic for patients affected by a sleep disorder.

### 2.2. Objective Excessive Sleepiness Related Measures

#### 2.2.1. Maintenance of Wakefulness Test

The MWT is the gold standard test to measure objectively chronic impaired vigilance (51). It measures precisely the ability to maintain cognitive performances with regards to wakefulness and arousal, to assess altered wakefulness. Concretely, it consists of asking the patients to resist sleeping when put to bed, with subdued light. The test is divided into four periods (10 a.m., 12 p.m., 2 p.m., and 4 p.m.), each separated by a 2 h gap. During the tests, EEG is recorded and lately scored by specialists, allowing to assess a sleep onset if one. The time between the test onset when the patients are put to bed and the lights are switched off, and the sleep onset (at least one epoch of any sleep stage) is called *MWT sleep latency*.

The reference medical measure mentioned in section 1 is the mean of the MWT sleep latencies across the four sessions. Each session has a maximal length of 40 min: sessions during which patients do not have slept are labeled with a MWT sleep latency of 40 min. Between iterations of the test, the patients have to stay awake and are free to perform any activity except sport. They have to stop smoking 30 min before the beginning of each nap and coffee, tea, or any stimulant substance consumption is prohibited. To simplify the problem into binary classification, we have labeled speakers as SL—i.e., having an altered vigilance—and NSL with a threshold of 19 min on the averaged MWT sleep latencies for each speaker, with respect to Doghramji et al. (52) and Sagaspe et al. (53).

#### 2.2.2. Multiple Sleep Latency Test

The MSLT is the gold standard measure of excessive sleepiness (51). It differs from the previous one in the dimension it measures: the MSLT is an objective measure of the propensity to sleep in a context of EDS, a symptom of numerous sleep disorders. Concretely, the MSLT procedure consists of asking the patients to take five naps a day, at 9 a.m., 11 a.m., 1 p.m., 3 p.m., and 5 p.m. The clinical procedure is the same as the MWT, and the duration between the beginning of the test and sleep onset is called *MSLT sleep latency*.

Each session has a maximal length of 20 min: sessions in which patients did not sleep are labeled with a 20 min MSLT sleep latency. On the contrary, patients having fallen asleep extend their naps until they have slept 15 min. The main difference between the MSLT and the MWT relies on the instructions, asking the subjects to “relax and drift off to sleep” whereas in the MWT they are asked to fight against sleep. Regarding the inter-sessions, the same instructions are given to the subjects as in the MWT procedure. More details about the MSLT procedure could be found in Littner et al. (34).

Relying on the interpretation of EEG by specialists to assess sleep onset, this medical test has been approved for numerous pathologies, including narcolepsy. For this latter pathology, the threshold to discriminate patients affected by narcolepsy against others is an averaged sleep latency of 8 min (54). In the same vein as a reference study on this test (51), we keep this threshold between SL speakers—i.e., patients having a pathological propensity to sleep—and NSL speakers in this study.

## 3. Corpora

This study is about corpora for automatic detection of sleepiness or excessive sleepiness. To our knowledge, few databases contain enough data for machine learning purposes (13). We, thus, decided to focus on four of them. Two of them have been used for international challenges on sleepiness estimation, and two of them have been recorded to design a machine algorithm that will be implemented in a virtual physician. On every one of them, we have computed statistics of the available data, dividing the speakers and samples between two classes (SL and NSL), following the threshold detailed in each part. When this piece of information is available for both the speakers and the samples, the two levels are labeled individually: the label of the speakers does not influence the label of the individual sample. Besides statistics on the data available on the corpora, we have also computed the total length of recordings, the mean length of the recordings, and, when the IDs of the patients were available, the mean number of samples per speaker.

### 3.1. Sleepy Language Corpus

Collected at the Institute of Psychophysiology, Düsseldorf, and the Institute of Safety Technology, University of Wuppertal, Germany, the SLC has been released in 2011 during the IS11 paralinguistic challenge (17) and comprises the recordings of 99 German speakers. Before the release of the SLEEP corpus in 2019, it has been the reference corpus for all the state-of-the-art systems (28). Even if the corpus is given with a train-development-test label for machine learning classification, we have chosen not to take into account this division to focus on medical information and corpus construction: the division being given for machine learning algorithm design, that is not the scope of this study.

#### 3.1.1. Population and Speech Tasks

The SLC is the aggregation of six partial sleep deprivation studies based on healthy subjects (55). The subjects are drafted from the general population and screened with the Pittsburgh Sleep Quality Index—(PSQI) (56)—to ensure that they are not affected by sleep disorders. Vocal samples were recorded either in a car environment [precisely described in Golz et al. (57)] or in lecture rooms. The vocal tasks performed by the subjects can be classified into five different categories:

- The reading of four simulated pilot-air traffic controller communication statements in English–labeled *Eng. reading*.
- The reading of the novel “Die Sonne und der Nordwind,” the German version of the story “the North Wind and the Sun”–labeled *Northwind*;
- The reading of simulated driver assistance system commands/requests–labeled *Reading*;
- Spontaneous speech (self-presentation);
- “Normal,” loud and smiling sustained vowel phonation.

**Figure 2:**
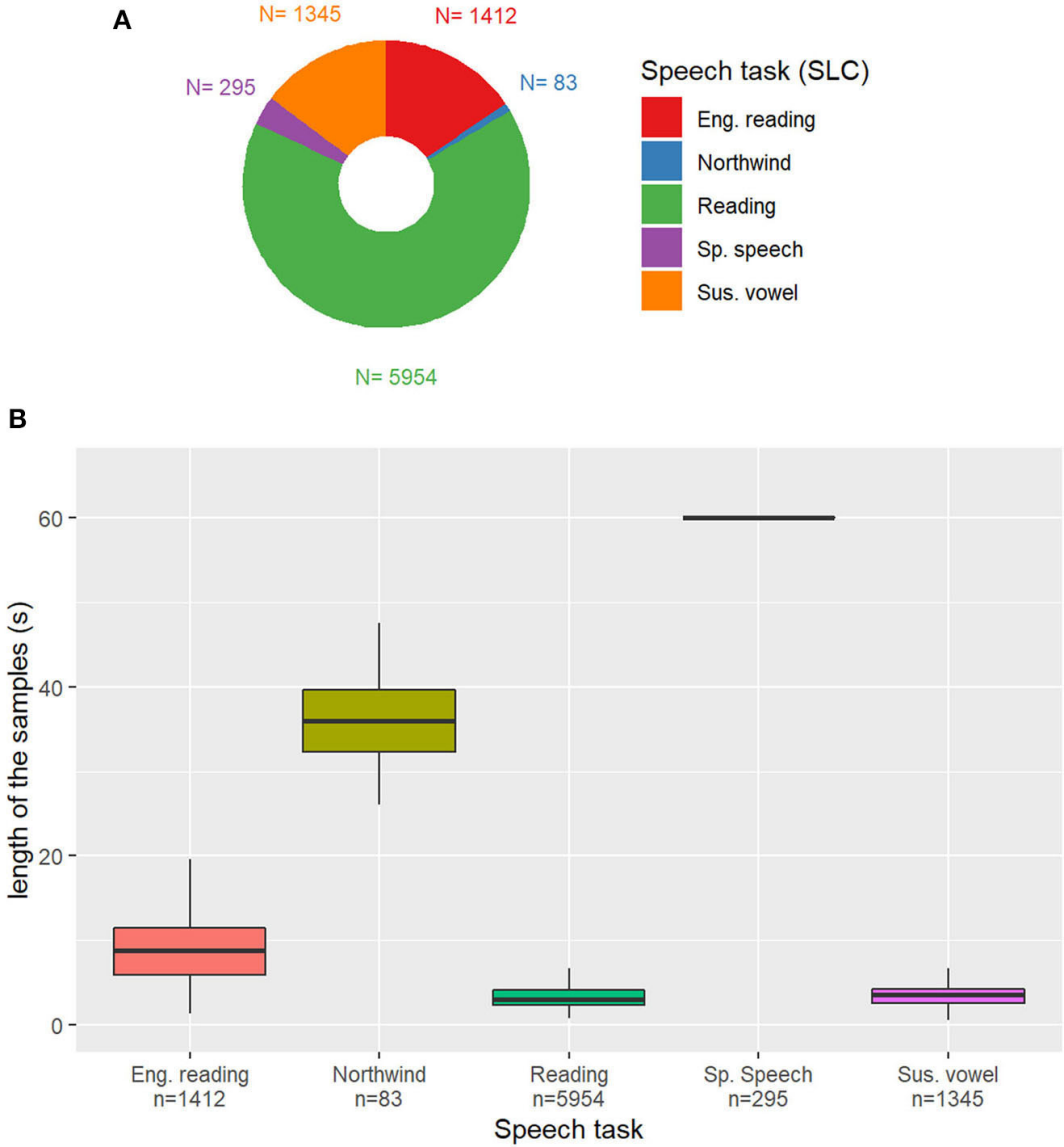
**(A)** Distribution of the speech tasks in the Sleepy Language Corpus (SLC). *Eng. Reading*: reading of simulated pilot-air traffic controller communication statements in English; *Northwind*: German version of the story “the North Wind and the Sun”; *Reading*: simulated driver assistance system commands/requests; *Sp. Speech*: self-presentation (spontaneous speech); *Sus. vowel*: sustained vowel. n: Number of samples in the category. **(B)** Duration of the samples depending on the task. Outliers removed for readability.

Except for the readings of flight control simulations that are in English, all the other samples are in German. The distribution of the samples among these classes is represented in Figure 2A.

#### 3.1.2. Sleepiness Label (KSS)

In the SLC, data are labeled with the 1–10 scale version of the KSS. The KSS value proposed in the SLC is slightly different from the medical version of the questionnaire that has been presented in section 2.1.1, as it is the average of three different KSS: one filled by the subjects, and two scored by external annotators, trained to evaluate sleepiness. Instead of the only subjective evaluation by the patient himself or herself, the proposed score is a mix between self-evaluation and behavior estimation of the sleepiness of the subject.

#### 3.1.3. Metadata

For this corpora, the other information associated with audio samples and sleepiness labels is the speaker ID and sex. No supplementary metadata concerning the participants is given. These data are presented in Table 1.

**Table 1:** Global statistics of the Sleepy Language Corpus (SLC).

**SPEAKER SCALE (SLC)**				
Men	43			
Women	56			
Samples/speaker (std)	91.8 (146.7)			
	**SL**	**NSL**	**TOTAL**	**sig.**
**SAMPLES SCALE (SLC)**				
Total length	6 h 6 min 9 s	15 h 10 min 39 s	21 h 16 min 48 s	
Avg. length of a sample (std)	7.0 s (11.3 s)	9.2 s (17.0 s)	8.15 s (15.3 s)	MW: ^****^
Samples	3,137	5,952	9,089	
Men	716	1,974	2,690	χ^2^: ****
Women	2,421	3,976	6,397	
KSS (std)	8.33 (0.57)	4.35 (1.78)	5.72 (2.41)	

*SL, Sleepy [Karolinska Sleepiness Scale (KSS)≥ 7.5)]; NSL, Non-Sleepy (KSS < 7.5); sig., significance of the statistical test; MW, Mann-Whitney's U-test*.

**** *p < 0.0001*.

### 3.2. SLEEP Corpus

Collected at the same location as the SLC, the SLEEP corpus has been released in 2019 during the IS19 paralinguistic challenge (18). The main advantage of this corpus relies on the great number of speakers (915) that were recorded, producing a total of 16,464 samples.

#### 3.2.1. Population and Speech Tasks

As no information is given on the recorded subjects, we assume that they are from the general population and have been screened with a PSQI. The subjects were recorded during sessions lasting between 15 min and 1 h. The speech material is divided between different reading passages and speaking tasks. Spontaneous speech has been recorded by asking subjects to comment on an event of their life (for example their last weekend or the best present they ever got) or to describe a picture. Unfortunately, no more precise information is given about the tasks in the article introducing the challenge, and this piece of information is not given in the database. In the same way, as for the SLC, the SLEEP corpus is given with divisions into training, development, and test subcorpora. The label annotation not being available in the test subset (this corpus is used for an international challenge), we focused on the train and the development subsets.

#### 3.2.2. Sleepiness Measure (KSS)

In the SLEEP corpus, the sleepiness measure is the same as in the SLC, except that annotations have been made with the 1–9 scale version of the KSS and that the averaged value is truncated to an integer.

#### 3.2.3. Metadata

Unfortunately, no metadata is available on this corpus: the only piece of information given in the corpus are the audio samples and their annotations with a KSS score. Only age is given by the article presenting the database (from 12 to 84 years, mean age: 27.6 ± 11 years). The data available on this corpus are presented in Table 2.

**Table 2:** Global statistics of the train and development subsets of the SLEEP corpus.

**SPEAKER SCALE (SLEEP CORPUS)**				
Men	551			
Women	364			
	**SL**	**NSL**	**TOTAL**	**sig.**
**SAMPLES SCALE (SLEEP CORPUS)**				
Total length	3 h 17 min 1 s	8 h 24 min 15 s	11 h 41 min 17 s	
Avg. Length of a sample (std.)	3.84 (0.66 s)	3.88 (0.64 s)	3.87 (0.64 s)	MW: ^*^
Nb. samples (train + dev)	3,081	7,811	10,892	
KSS (std)	7.61 (0.66)	4.07 (1.4)	5.07 (2.01)		

*SL, Sleepy (KSS≥ 7.5); NSL, Non-Sleepy (KSS < 7.5); sig., significance of the statistical test; MW, Mann-Whitney's U test*.

* *p < 0.05*.

### 3.3. Maintenance of Wakefulness Test Corpus

To our knowledge, the MWTc was the first attempt to elaborate a large voice corpus annotated with objective sleepiness, measured by EEG. It has been recorded at the Sleep Clinic of Bordeaux's University Hospital on 75 patients undertaking an MWT. Due to numerous biases and incomplete data for a large number of speakers, this database has not been released. Nevertheless, it has paved the way for other corpora, such as the MSLTc, presented in section 3.4. Moreover, it presents interesting characteristics to nourish the later discussions about methodology.

#### 3.3.1. Speech Tasks

Before each iteration of the test, the patients are asked to read a text, that is either a summary of a scientific article or a fable. The recordings are made with a microphone integrated into a webcam, at a distance of approximately 30 cm of the mouth of the patient. A brief description of these texts (number of words and mean duration of the corresponding recordings) is presented in Table 3A. The speakers of the MWTc present signs of EDS and are suspected to be affected by pathologies causing a drop in attention during the day. As data are missing for numerous patients, statistics made at the sample level are separated from speakers statistics, the latter being made on the 57 speakers for which we have the four values of MWT, to give a meaningful value for the averaged MWT value.

**Table 3:** Brief description of the texts read by the patients of the Maintenance of Wakefulness Test Corpus (MWTc) **(A)** and Multiple Sleep Latency Test corpus (MSLTc) **(B)** corpora.

**Session**	**Text**	**Length (Nb. words)**	**Mean length±std**
**(A) MWTc**			
1	Rats holding court	196 words	75.5 ± 15.0 s
2	Why you should not drink sea water to stay hydrated	282 words	112.0 ± 18.4 s
3	Providential glasses	163 words	60.1 ± 12.0 s
4	Science gives you an excellent reason to eat chocolate !	278 words	123.5 ± 26.2 s
**(B) MSLTc**			
1	Text 1	231 words	77.9 ± 11.6 s
2	Text 2	235 words	79.3 ± 12.2 s
3	Text 3	228 words	72.7 ± 10.8 s
4	Text 4	221 words	77.2 ± 12.2 s
5	Text 5	257 words	80.6 ± 12.4 s

#### 3.3.2. Sleepiness Label (MWT)

The samples of the MWTc are labeled in two different ways: with the averaged MWT sleep latency of the speaker, for speaker trait estimation; and with the individual MWT sleep latency, for speaker state estimation. The individual sleep latencies are not medically validated but can be an objective marker of short-term wakefulness variations across the sessions. The independent samples are labeled into SL and NSL with the same 19 min threshold as the averaged MWT sleep latency, independently from the label of the speaker having vocalized it.

#### 3.3.3. Metadata

Supplementary data contains the ID, age, sex, Body mass index (BMI), diagnostic of speakers for Obstructive Sleep Apnea (OSA), and the ESS is filled by each patient. The Toronto Hospital Alertness Test (43) and the Cartoon Faces Sleepiness scale (58) have also been collected, but due to too few gathered values, they are not reported in this article. The data available on this corpus are presented in Table 4.

**Table 4:** Global statistic of the MWTc on the speakers and the samples levels.

	**SL**	**NSL**	**TOTAL**	**sig**.
**SPEAKERS SCALE (MWTc)**				
Spk.	11	46	57	
Age (std) years	45.9 (15.6)	46.2 (15.9)	46.14 (15.72)	MW: n.s.
BMI (std) kg/m^2^	30.2 (4.4)	18.5 (13.0)	20.8 (12.4)	MW: ^**^
OSA	9	25	34	χ^2^: n.s.
W.o. OSA	2	20	22	
Men	10	29	39	χ^2^: n.s.
Women	1	17	18	
MWT (std) minutes	10.36 (4.9)	34.1 (7.21)	29.53 (11.64)	
ESS (std)	13.44 (4.53)	10.93 (5.24)	11.38 (5.17)	MW: n.s.
**SAMPLES SCALE (MWTc)**				
Total length	2 h 04 min 14 s	5 h 08 min 36 s	7 h 12 min 51 s	
Avg. Length (std)	1 min 33 s (31.9 s)	1 min 30 s (31.3 s)	1 min 32 s (31.7 s)	MW: n.s.
Samples	83	199	282	
Men	63	126	189	χ^2^: n.s.
Women	20	73	93	
MWT (std) minutes	6.37 (4.12)	38.52 (4.37)	29.06 (15.3)	

*SL: Sleepy (avg. MWT ≤ 19 min and MWT ≤ 19 min respectively for speaker and sample levels). NSL, Non-Sleepy; sig., significance of the statistical test; MW, Mann-Whitney's U test; n.s., not significant,*

** *p < 0.01*.

### 3.4. Multiple Sleep Latency Test Corpus

The MSLTc has been elaborated following the MWTc. Also recorded at the Sleep Clinic of the Bordeaux's University Hospital, this corpus follows the same goal as the MWTc, i.e., linking objective measures of hypersomnolence and vocal recordings.

#### 3.4.1. Population and Speech Tasks

The methodology to collect the data of the MSLTc is thoroughly described in Martin et al. (13). It consists of the recordings of 106 patients taking a medical test for diagnosis or follow-up at the Sleep Clinic of the Bordeaux University Hospital. This test is a MSLT, consisting of asking the patient to take a nap five times a day. Before each nap, the subjects are recorded reading a text of approximately 200 words, and fill a KSS. The subjects are generally sat down at their desks or on their bed. No recording has been done with lying down patients. The read texts are extracts from “Le petit prince” of Antoine de Saint-Exupéry. Basic characteristics of these texts (number of words and mean duration of the corresponding recordings) are presented in Table 3B.

#### 3.4.2. Sleepiness Label (MSLT)

In the same way, as in the MWTc, samples are labeled with both averaged MSLT sleep latency and individual sleep latencies. The same threshold of 8 min is used to binarize averaged MSLT sleep latencies and individual sleep latencies independently. The individual MSLT sleep latencies are not medically validated but can be an objective marker of short-term propensity to sleep across the naps.

#### 3.4.3. Metadata

This corpus contains numerous Supplementary Materials about the speakers: physical measures (height, weight, BMI, neck size), age, sex, cigarettes and alcohol dependency, fatigue, insomnia, and multiple sleepiness-related questionnaires.

**Figure 3:**
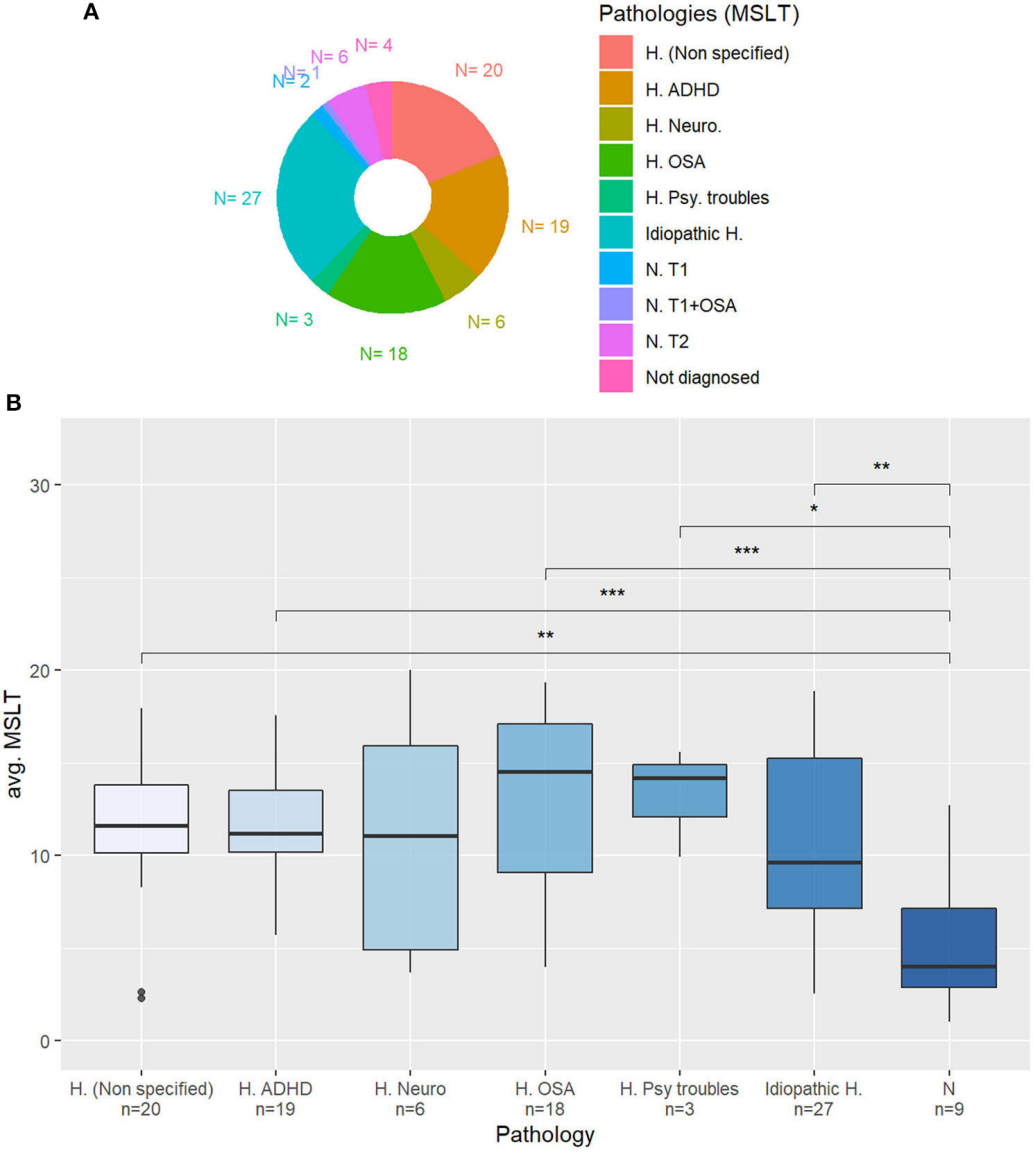
**(A)** Diagnosed pathology for the patients of the Multiple Sleep Latency Test corpus (MSLTc). *H. (Unspecified)*: Non-specified hypersomnia (complementary exams needed); *H. ADHD*: Hypersomnia in an attention-deficit hyperactivity disorder context; *H. Neuro*: Neurological hypersomnia; *H. OSA*: Hypersomnia with Obstructive Sleep Apnea; *H. Psy. Troubles*: Hypersomnia accompanied by psychiatric troubles; *Idiopathic H*.: Idiopathic Hypersomnia; *N. T1*: Type 1 Narcolepsy; *N. T1+OSA*: Type 1 Narcolepsy + OSA; *N. T2*: Type 2 Narcolepsy; *Not diag*.: Not yet diagnosed. **(B)** Averaged MSLT sleep latencies depending on the pathology. N: Narcolepsy. Mann-Whitney's *U*-test: *p < 0.05; **p < 0.01; ***p < 0.001.

In this study, we will restrain to measures that can be compared with the other corpora: sleepiness measures, age, sex, BMI, ESS, and KSS distribution are presented in Table 5. To our knowledge, this corpus is also the first to collect the pathologies of the patients whose voices are recorded, represented in Figure 3A.

**Table 5:** Global statistics of the MSLTc on the speaker and the sample levels.

	**SL**	**NSL**	**TOTAL**	**sig**.
**SPEAKERS SCALE (MSLTc)**				
Spk.	28	78	106	
Age (std) years	33.5 (15.9)	36.7 (13.5)	35.9 (14.2)	MW: n.s.
BMI (std) kg/m^2^	24.1 (4.7)	24.3 (5.6)	24.2 (5.3)	MW: n.s.
Men	15	28	43	χ^2^: n.s.
Women	13	50	63	
avg. MSLT (std) minutes	4.84 (2.05)	13.53 (3.22)	11.23 (4.85)	
avg. KSS (std)	4.37 (1.22)	4.47 (1.28)	4.45 (1.26)	MW: n.s.
ESS (std)	15.91 (5.68)	14.19 (4.53)	14.65 (4.89)	MW: ^*^
**SAMPLES SCALE (MSLTc)**				
Total length	4 h 30 min 9 s	6 h 54 min 44 s	11 h 24 min 54 s	
Avg. length of a sample (std)	1 min 17 s (12.0 s)	1 min 17 s (12.2 s)	1 min 17 s (12.1 s)	MW: n.s.
Samples	210	320	530	
Men	100	115	215	χ^2^: ^**^
Women	110	205	315	
MSLT (std) minutes	4.55 (2.21)	15.62 (4.28)	11.23 (6.51)	
KSS (std)	4.58 (1.89)	4.36 (1.92)	4.45 (1.91)	MW: n.s.

*SL, Sleepy (avg. MSLT ≤ 8 min and MSLT ≤ 8 min respectively for speaker and sample levels); NSL, Non-Sleepy; sig., significance of the statistical test; MW, Mann-Whitney's U-est; n.s., not significant;*

* *p < 0.05*,

** *p < 0.01*.

## 4. Comparison between corpora

In the following, Mann-Whitney statistical tests will be referred to as MW.

### 4.1. Speech Tasks and Audio Samples Measures

#### 4.1.1. Length Depending on the Sleepiness Class

On both the SLC and SLEEP corpora, the NSL samples are significantly longer than the SL ones (MW, SLC: *p* = 6.5 × 10^−10^, SLEEP: *p* = 0.03). On the contrary, on the MSLT and MWT corpora, the length of the samples is the same in the two sleepiness classes.

#### 4.1.2. Length Depending on the Task/Iteration

The length of the samples depending on the task on the SLC is represented in Figure 2B. Except for samples containing spontaneous speech, the majority of samples are shorter than 10 s, because of the vast representation of reading and sustained vowels samples. Some reading tasks are longer than others, with English language readings being on average 8.7 s long and Northwind readings being at least two times as long, with a mean duration of 36.6 s.

**Figure 4:**
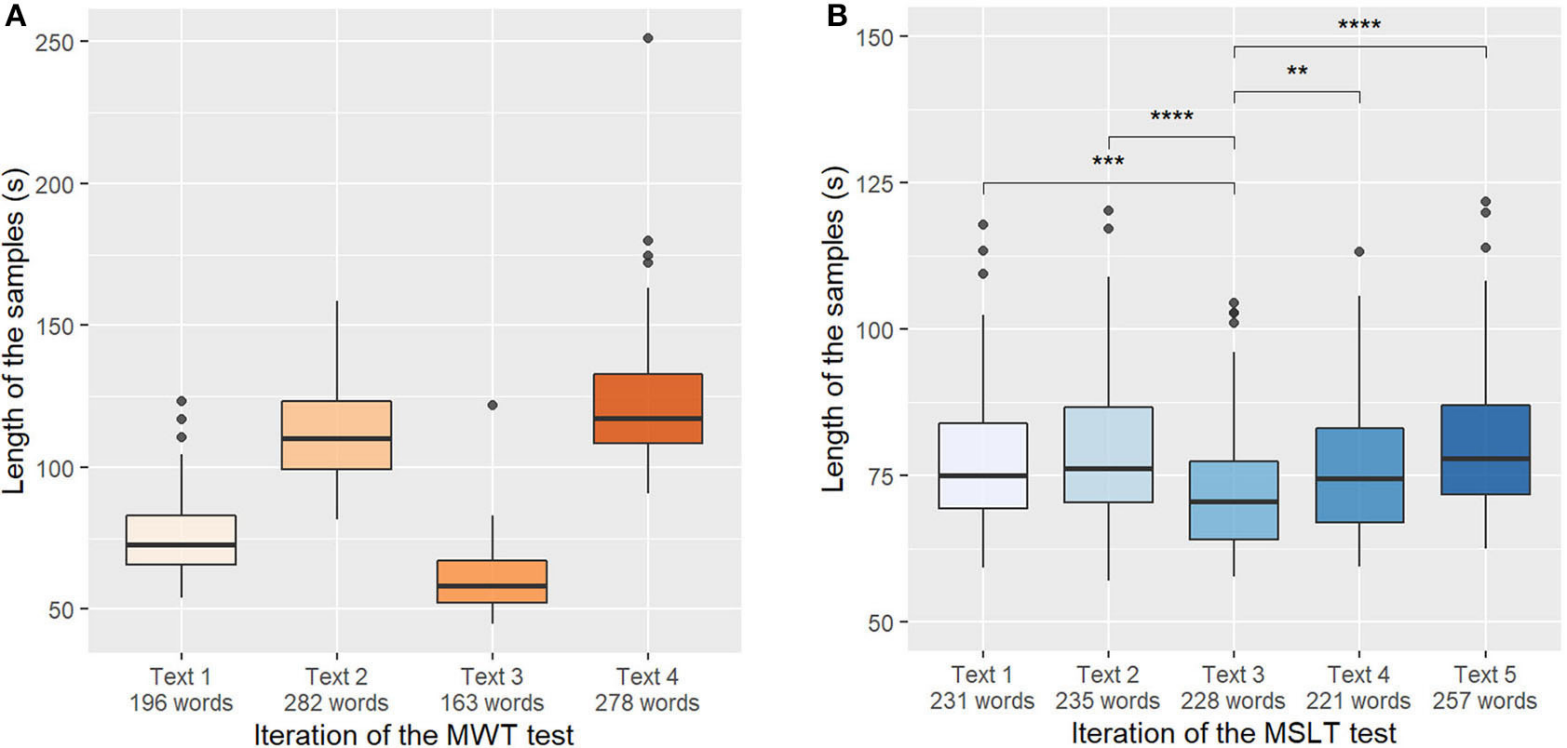
**(A)** Length of the samples depending on the iterations of the Maintenance of Wakefulness Test (MWT). Significance masked for readability. **(B)** Length of the samples depending on the iterations of the Multiple Sleep Latency Test (MSLT). Mann-Whitney's *U*-test: **p < 0.01; ***p < 0.001; ****p < 0.0001.

The length of the samples depending on the read text (and the iteration of the test) of the MWTc and MSLTc corpora are represented in Figures 4A,B. On the MSLTc, all the recordings have similar sizes (approximately 75 s) except for the third one, which is shorter than all the others. Already studied in Martin et al. (13), this observation is not only due to a difference of lengths of the texts, but it has been linked on this iteration to a diminution of the KSS—i.e., an augmentation of the alertness of the speakers.

On the contrary, on the MWT database, all the sessions have different lengths. This difference mainly comes from the fact that there are two types of texts in this database: fables during the first and third iterations, and popular science articles for the second and fourth iterations. These texts have not the same number of words: fables have a respective length of 187 words and 161 words for the first and third iteration, whereas popular science articles have a respective length of 269 and 286 words.

#### 4.1.3. Number of Samples per Speaker

**Figure 5:**
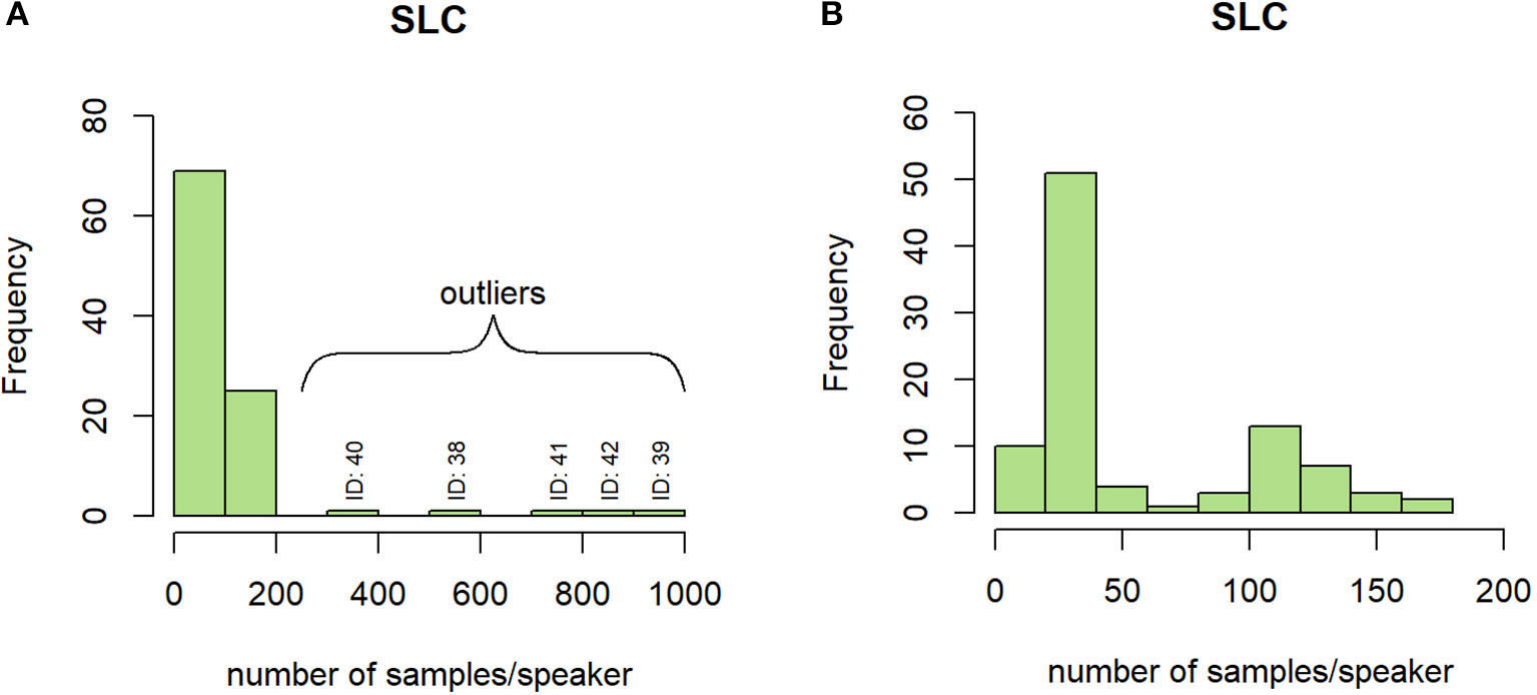
Histogram of the number of samples per speaker on the SLC with **(A)** and without **(B)** outliers.

On the SLC, depending on the sub-experiment they took part in, the subjects were recorded between 3 and 909 times, showing an important disparity. In Figure 5A, five speakers separate from the main distribution: the statistics concerning this metric have been re-computed without these five outliers, changing the mean value from 91 samples to 61 samples per subject. The resulting histogram is represented in Figure 5B.

In the SLEEP corpus, the recording sessions have a length between 15 min and 1 h: the number of samples per speaker could be unbalanced when comparing speakers being recorded during a session of 15 min and those who are recorded during a 1-h session.

Finally, in the MSLT and MWT corpora, the number of samples per speaker is set by the iterations of the medical test.

### 4.2. Medical Labels

#### 4.2.1. MWT and MSLT Sleep Latencies

**Figure 6:**
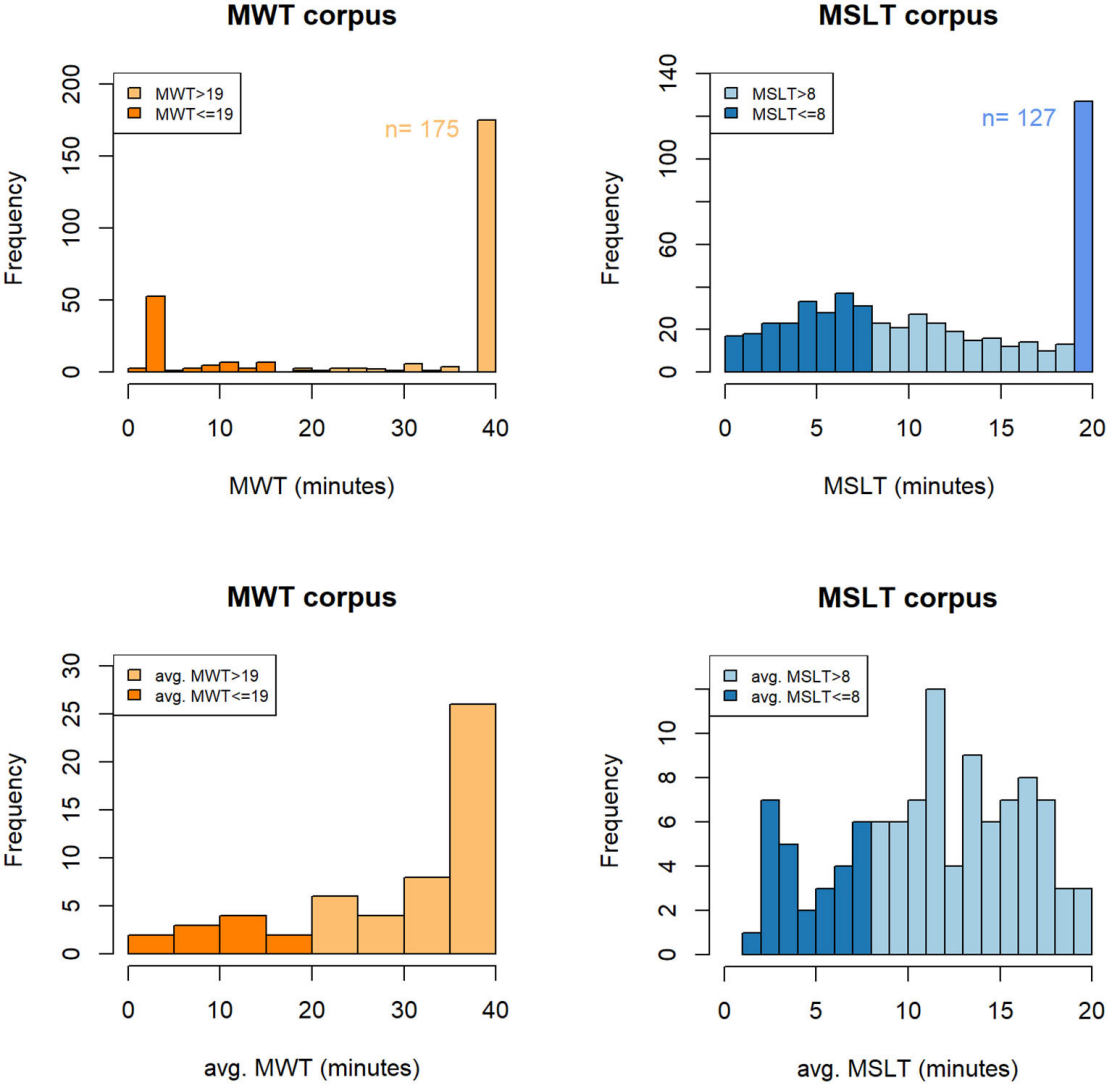
Histogram of the objective measures of the short-term **(A,B)** and the long-term **(C,D)** sleepiness on the MWTc and MSLTc. MWT, sleep latency to the Maintenance of Wakefulness Test; MSLT, sleep latency to the Multiple Sleep Latency Test; avg. MWT, average sleep latency across the four sessions of the Maintenance of Wakefulness Test; avg. MSLT, average sleep latency across the five naps of the Multiple Sleep Latency Test; n, number of samples corresponding to saturation.

On the MWT and MSLT iteration values, a sleep latency saturation is observed for patients that do not fall asleep and that are assigned to the maximal value of the test (40 min for the MWT and 20 min for the MSLT). We employ in this study the term “saturation” as these values not only unbalance the global distributions (cf Figures 6A,B), but they also represent an important part of the two corpora. Indeed, they represent 62 and 24% of the samples in the MWTc and the MSLTc, respectively.

#### 4.2.2. MWT and MSLT Averaged Sleep Latency

The averaged sleep latencies of the MWT and MSLT are represented in Figures 6C,D. On the MWTc, the prominence of the iteration sleep latencies labeled as 40 min seems to have a consequent impact on the distribution of the averaged sleep latencies, which has the same saturation on the 40 min values. On the contrary, on the MSLTc, few speakers have all their sleep latencies equal to 20 min, leading to a smoothing of the averaged MSLT sleep latencies and the disappearance of the saturation.

#### 4.2.3. Karolinska Sleepiness Scale

**Figure 7:**
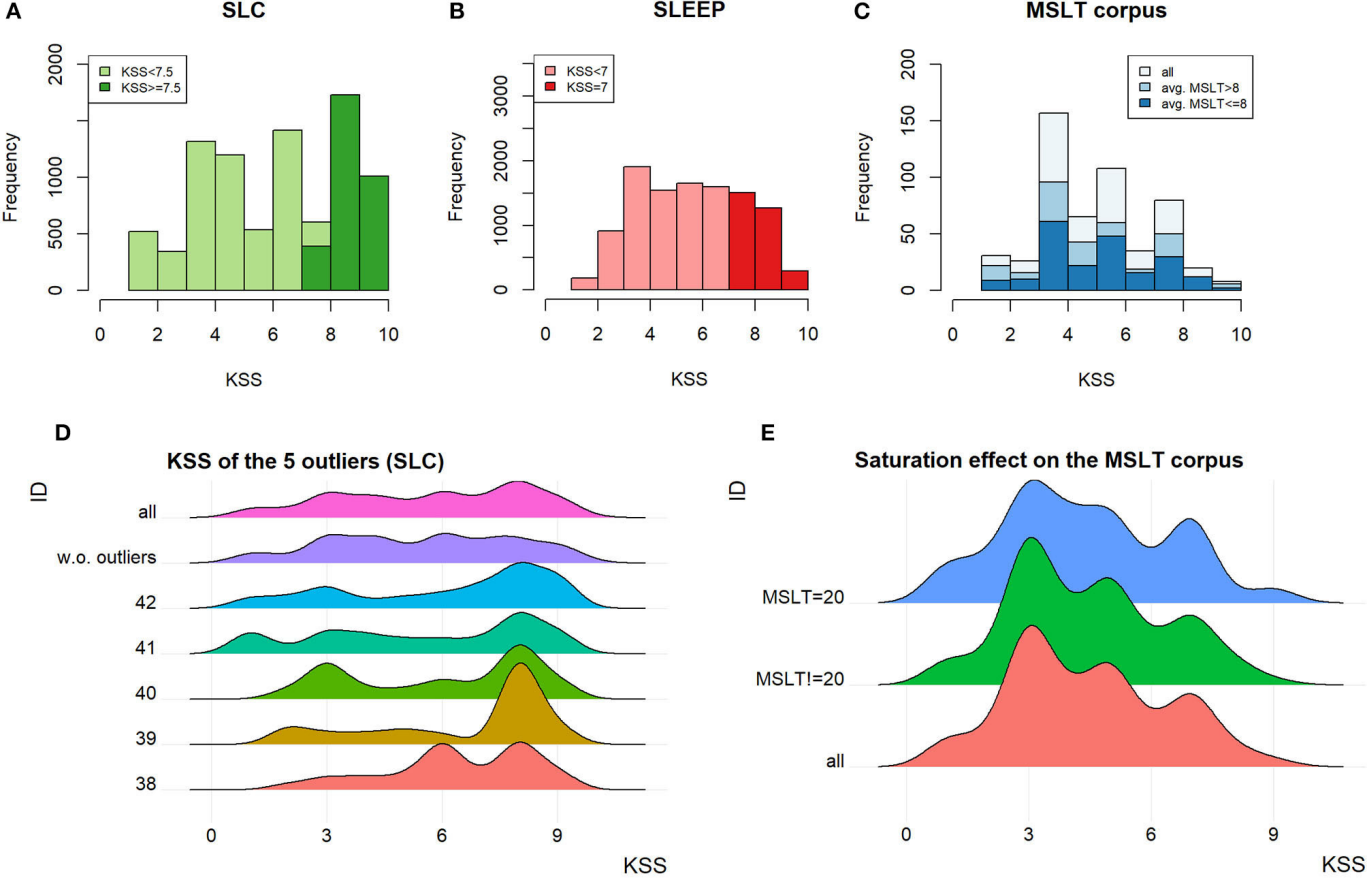
**(A–C)** KSS distribution on the SLC, the SLEEP corpus, and the MSLTc. **(D)** KSS distribution of the outliers on the SLC and their impact on the distribution of the whole SLC. **(E)** Influence of the saturation on the KSS in the MSLTc.

The KSS on the SLC, SLEEP corpus, and MSLTc are, respectively, plotted in Figures 7A–C. These three measures are different: on the SLC, it is the average of three 1–10 scale KSS questionnaires, one filled by the patient, the other two being filled by external annotators; on the SLEEP corpus it is the same procedure with the 1–9 scale KSS with a truncated mean; finally, on the MSLTc, a 1–9 scale KSS is filled only by the patient.

On the SLC, the speakers outlying the number of samples per speaker also influence the KSS distribution. Indeed, the difference observed on the KSS distribution of the whole corpus with and without these outliers, represented in Figure 7D, is significant (MW, *p* < 10^−13^). This difference leads to an important change in the balance between SL and NSL: with the outliers, SL samples represent 34% of the 9,089 samples; whereas, without them, this ratio falls to 27.5% (1,589 samples) of the 5,776 total remaining samples.

The distribution of the KSS on the MSLTc differs from the two other corpora by the over-representation of the uneven scores compared with the even ones. The item “3–Alert” is the most represented, followed by “5–neither alert nor sleepy” and “7–sleepy, but no effort to stay awake.” Contrary to the exclusion of the speakers having been recorded too many times on the SLC, the exclusion of the samples corresponding to the 20 min saturation value on the MSLTc does not have any influence on the KSS distribution. Indeed, the two distributions (with and without these samples) plotted in Figure 7E do not show major differences, which is confirmed by a statistical test (MW: *p* = 0.91).

#### 4.2.4. Correlation Between KSS and MSLT

We have first hypothesized that the absence of correlation between the MSLT sleep latencies and the KSS (Spearman's ρ between KSS and MSLT iteration value, with saturation: ρ = −0.034) were due to the saturation. This hypothesis is contradicted by the negligible augmentation of the correlation factor when keeping out the samples causing it (ρ = −0.042). Therefore, there seems to be an intrinsic difference between the MSLT iteration value taken individually and the KSS to evaluate instantaneous sleepiness, which is discussed in section 5.5.

#### 4.2.5. Epworth Sleepiness Scale

On the MWTc, the ESS distribution is almost significantly different across the two sleepiness categories (MW, *p* = 0.067). On the MSLTc, it differs from the SL class compared with the NSL class (MW, *p* = 0.04). Moreover, this metric is almost anti-correlated with the averaged MSLT value (Spearman's ρ = −0.186, *p* = 0.057): it is in line with the fact that ESS measures in a subjective way the same phenomena as MSLT (high propensity to sleep, as shown in Figure 1).

### 4.3. Metadata

#### 4.3.1. Sex

In the SLC, the ratio between men and women is almost balanced on the speaker scale, but an important significant imbalance is observed between samples containing the voice of women and men (χ^2^, *p* < 2.2 × 10^−16^). This imbalance is mainly due to the few speakers that produced a lot of samples (referred to before as *outliers*): without these five speakers, the samples are almost balanced (3,084 performed by Women and 2,690 by Men) and their distribution among SL and NSL classes is balanced (χ^2^, *p* = 0.16).

In the MWTc, the 57 speakers are distributed among 39 men and 18 women. The distribution of sex between sleepy and non-sleepy speakers seems to be imbalanced, but a χ^2^ test processed on this distribution shows independence between sex and sleepiness class on both the speaker scale (χ^2^, *p* = 0.15) and the sample level (χ^2^, *p* = 0.056).

Contrary to the MWTc, there is an imbalance of sex across the SL and NSL classes in the MSLTc. It is not proven significant at the speaker level (χ^2^, *p* = 0.16), but it is significant at the sample level (χ^2^, *p* = 9.6 × 10^−3^).

#### 4.3.2. Age

The speakers of the MWTc (mean age 46.3 years) and those of the MSLTc (mean age 35.9 years) are older than the speakers of the SLC or SLEEP corpus, with respective mean ages of 24.9 and 27.6 years. In the two first corpora, only few differences are observed between SL and NSL patients (MW, *p* = 0.93 for the MWTc, *p* = 0.96 for the MSLTc).

Their differences with the SLEEP and the SLC can be explained by the fact that the MSLT and MWT corpora do not contain recordings from juvenile patients, and that the recorded patients are drafted from a pathological population in which the prevalence of the pathology is linked with age.

#### 4.3.3. Body Mass Index

The significant imbalance of BMI observed between SL and NSL classes in the MWTc corpus (MW, *p* = 0.003) may not be exclusively the consequence of the unbalance between men and women in this corpus. Indeed, in the general French population, the average BMI is 25.8 kg/m^2^ with only a slight difference between men (25.8 kg/m^2^) and women (25.7 kg/m^2^) (59). The values of these general populations are higher than the observed values in the MWTc.

On the contrary, on the MSLTc, the BMI is closer to the precedent normative values with a mean BMI of 24.2 kg/m^2^, with no significant differences between the SL and the NSL patients (MW, *p* = 0.89).

#### 4.3.4. Pathologies

In the MWTc, the only available medical diagnosis is OSA that is balanced between the two sleepiness classes (χ^2^, *p* = 0.21). This factor is still to take into account when estimating the sleepiness from voice as these patients could have a different shape of the superior airways.

In the MSLTc, the distribution of the averaged MSLT sleep latencies depending on the diagnosed pathologies is plotted in Figure 3B. Merging the classes “NT1” and “NT2” into a unique “Narcolepsy” category, the patients affected by this disease have significantly lower averaged sleep latency in the MSLT than almost all the other subjects. Regarding the other categories, all the other patients have equivalent averaged sleep latency.

## 5. Guidelines

The following sections discuss the different questions that any corpus designer could ask when designing a database, aiming at one or the other paradigm.

### 5.1. Choice of the Subjects

#### 5.1.1. Healthy Subjects, General Population, Patients

When designing a corpus, one of the first questions that are raised is the population to be recorded. Regarding the tasks of estimating sleepiness or excessive daytime sleepiness through voice, two populations should be considered: on the one hand, included subjects should be patients when the task is the follow-up of patients; on the other hand, subjects should be picked from the general population when the task is the estimation of a “general” sleepiness state. On the MSLT and MWT corpora, the recorded subjects are patients of the Sleep Clinic of Bordeaux, affected by excessive sleepiness.

On the SLC and SLEEP corpus, the recorded speakers are drafted from the general population and screened with a PSQI questionnaire to ensure they are healthy regarding sleep disorders. The difference between general and healthy populations is a key point in this study: one can be drafted from the general population and suffering from undiagnosed sleep diseases. However, the notion of “healthy subject” does not stop at the primary outcome of a study: ideally, they should be healthy (i.e., negative to a diagnostic—or a screening questionnaire) not only regarding sleep disorders but also regarding all the other cofactors that could influence the measured phenomena, such as anxiety, depression, and fatigue.

#### 5.1.2. Balancing the Dataset

Balancing the dataset is both important for ethical reasons (60) but also to ensure that vocal characteristics or measured phenomena are independent of the characteristics of subjects such as sex, age, and BMI. However, balancing all these characteristics is technically impossible. To shrug these biases off databases, we propose two directions. First, the inclusion of these metadata in databases, such as the machine learning engineers using them could take them into account. Second, a difference should be made depending on the objective of the study. To study a phenomenon in the general population, the best practice is to include enough subjects so that these biases might be randomized. On the contrary, when working with a population affected by a disorder having a greater prevalence in a specific subset of the population, restraining to this subgroup allow a good generalization of the concepts (61).

#### 5.1.3. Reading Level and Oral Communication

In the case of reading text, reading them out loud is a process involving multiple neuro-lingual and neuro-motor processes (62), that can be affected by both sleepiness and the reading capacities of the reader. To ensure that all the information extracted from the recordings to design algorithms is impacted only by the neuro-psychiatric measure (sleepiness in this case) and not by the speaker reading capacities, corpora designers should exclude the speakers presenting reading troubles, or that do not have sufficient reading abilities. This is the case, to our knowledge, of only one previous work (15) that has paid attention to selecting the speakers based on their reading level in collaboration with speech therapists.

The same precaution should apply when dealing with spontaneous speech, ensuring that the hesitations and all the other extracted vocal features are linked to sleepiness, not by the emotional state or pathology of the speaker. For example, patients affected by dysphonia, anxiety, or vocal tract problems should have a voice different from the other speakers of the database, not based on their sleepiness but on other pathologies that affect their vocal production. While a lot of attention is paid to the medical condition of speakers in corpora, we encourage database designers to take into account this key point in the future.

#### 5.1.4. Conclusions and Recommendations for Choice of Subjects

- **Choice of the population**
  - For following-up patients, a pathological population with controlled comorbidities is required;
  - Recording healthy subjects seem more fitted for sleepiness detection in the general population, but they require more exigent inclusion and exclusion criteria than the general population. This could make the recruitment of subjects difficult but avoids interference between the measured phenomena and comorbidities.

- **Speakers characteristics**
  - When working on the general population, the number of subjects should be high enough to randomize the effect of co-factors.
  - When working with patients, they should comply with the specificity of the pathologies.
  - In both cases, co-factors should be included in the databases.

- **Disorders affecting reading or voicing capacities**
  - Subjects should not be affected by pathologies interfering with their abilities to read a text or talk naturally.

### 5.2. Design of the Recording Session(s)

When the population to be included in the study has been selected, multiple recording configurations are still possible.

#### 5.2.1. Equality of Recordings per Speaker

First, the best practice when constructing a database is to ensure that the same number of samples is recorded for each speaker, in the same conditions. As seen in section 4.1.3, all the speakers of the SLC have not been recorded the same number of times. This results in substantial changes in KSS and sex distribution between the two sleepiness classes. In this corpus, sex and KSS were the only available data about the speakers, but the over-representation of these speakers may also have created biases on unmeasured data, such as age, and BMI.

#### 5.2.2. Number of Recordings per Speaker

We have argued that the best practice is to record each speaker the same number of times, but how many recordings per speaker have to be recorded?

A recording of the voice necessarily contains both the expressions of the traits of the speaker (sex, age, long-term sleepiness, …) and those of the state of the speaker (mood, fatigue, short-term sleepiness, circadian cycle, …). Consequently, isolating one phenomenon expression from the other requires either measuring both and taking into account the undesired one when estimating the interesting one; or randomizing the undesired factor. Both strategies imply multiple measures.

In the MSLT and the MWT procedures, iteration sleep latencies are measured at different standardized times before being averaged, to estimate the traits of speakers independently from their short-term variations states: the state of the speaker varies across the recordings, whereas its traits remain invariant.

Regarding speaker state estimation, the same procedure has been set up in the MSLTc with the collection of the KSS: regularly timed measures allow to estimate the traits of the speaker that remain constant before keeping variations from these to estimate speaker states. Collecting regularly timed measures of the KSS has also been set up in the car simulator part of the SLC (57): between 45 min driving sessions, the subjects performed various vocal tasks and filled a KSS.

However, except when designing the collection of data upon another study, it seems complicated to record subjects along long periods of time needed to record subjects in different states. To avoid the creation of an intrinsic link between the speaker identity and the labels, due to the over-representation of speakers by samples with few state variations, Huckvale et al. (29) suggests investing efforts on recording numerous speakers fewer times. This randomizes speaker traits and allows a correct estimation of speaker states, independently from the characteristics of the speaker. However, the drawback of this method is that a sufficient amount of subjects is required to randomize the characteristics of the speakers, which could be difficult regarding the inclusion and exclusion criteria on the subjects (cf section 5.1).

#### 5.2.3. Recording Session Length

In the SLEEP corpus, the recording sessions have a length between 15 min and 1 h but the samples have a maximum length of 5 s: the recordings have been sliced to augment the number of samples in the corpus. This has some advantages, such as making the most of the presence of each speaker by recording them on the longest possible sessions or having large corpora including only a few speakers. But doing so has mainly three drawbacks.

First, psychometric questionnaires which are used to label data are designed to embrace a state during a specific period. As the state of the speaker may vary quickly, it is possible to make multiple measures of the same speaker presenting different states in a small period of time. However, regarding the KSS, this questionnaire is not originally designed and medically validated for repeated measures (63). Reiterating the filling of a KSS questionnaire after less than 10 min could give different scores, not because of changes in the sleepiness of the speaker but only because the questionnaire has not been designed and validated to do so. One filling of a KSS by an interval of 10 min should be sufficient to assess the sleepiness state on the whole temporal slice and to collect enough data per session.

Second, the number of samples would not be the same for all the speakers, over-representing some speakers and other biases linked to the recording session. Moreover, it goes in the opposite direction of the discussion in the previous paragraph. Finally, recording sessions of this length induce both vocal fatigue (64) and cognitive fatigue that could affect both voice production and sleepiness estimation. Even if slicing samples can be done—with some precaution such as the minimum length to slice, discussed in section 5.4—the length of the session should be kept reasonable and equal among the participants.

#### 5.2.4. Recordings Location

After having discussed the design of the recording sessions, the question of the location of the recording is still to be answered. The literature mentions three different recording set-up: in a hospital room (13), in a car simulator (57), and in a quiet room (18). Two methodological choices are opposing. First, recording in condition as near as possible from the final application, such as the driving simulator for example. However, this recording environment creates a bias on the feelings of the patients, limiting the exploitation of the study.

On the other side, logistic facilities tend to favor hospital conditions. Admittedly, they can create stressful conditions for people not being used to being hospitalized (65). But they also offer easy access to EEG, the only way to measure objective sleepiness, and perfectly controlled conditions: the participants are treated equally, have their meal at the same time, and their sleep the night before the medical test is controlled. All these advantages tend to encourage recordings in hospital conditions in the first place, before extending the problem to ecological conditions.

#### 5.2.5. Recording Quality

The quality of the recordings can be affected by multiple sources of noise in both realistic (noise due to the car simulator or from car traffic for example) and hospital conditions, even in sleep clinics (due to ventilation noise for example). As a consequence, a quiet and non-reverberating room should be favored.

The other aspect impacting the quality of the recordings is the chosen recording device, which takes over the previous dichotomy between realism and control over the collected data. While an increasing number of studies are based on smartphones recordings [e.g., Huang et al. (66)], it could be feared that acoustic features extracted from these recordings may suffer from the low recording quality of these devices.

A recent study did, however, not find differences in performance on Parkinson's disease identification from voice, comparing high-quality recording and smartphone data (67). Moreover, the features extracted from the speech are not limited to acoustic parameters: diverse linguistic features also give useful hints on diverse pathologies (15, 68) and are less affected by the quality of the recording than the acoustic ones.

#### 5.2.6. Conclusion and Recommendations on the Recording Session

- **Multiplying the measure**
  - Either multiplying the measures of the same speaker in different states for speaker trait estimation, or multiplying the number of recorded subjects to randomize the traits of the speaker for speaker state estimation.

- **Length of the recording session**
  - For both biases and fatigue reasons, we encourage the standardization and the limitation of the length of the recording session.

- **Recordings location**
  - We encourage to favor in hospital rooms first, then to extend to location as close as possible to the final application.

- **Recordings quality**
  - We encourage to process the recordings in calm and non-reverberating environments,
  - The choice between smartphone or high-quality microphone recordings depends on the features used in the study and the need for realism or at-home deployment. However, it is still possible to deteriorate audio recorded from a high-quality microphone to make it imitate smartphone recordings if needed, while the opposite is not possible.

### 5.3. Material to Record Voice

Having chosen the temporal sequence of the recording, the task on which recording speech has to be chosen.

#### 5.3.1. Spontaneous Speech vs. Reading

Being during the interaction with a virtual or real physician or during a telephonic call, spontaneous speech can be easily recorded in ecological conditions. As a consequence, designing a corpus based on spontaneous speech seems the most natural way to record the voice of the participants for ecological usage. Nevertheless, this paradigm suffers from three main drawbacks. First, when elaborating machine learning algorithms, the voice of the subject has to be the least possible polluted by his or her emotional state, to prevent the system to discriminate emotions instead of sleepiness states. Open questions implying memories or the emotions induced by a painting—as is the case in the SLEEP corpus—change the emotional state of the speaker. Second, as stated in a previous study (69), this paradigm suffers another disadvantage: if the questions are identical, the samples have neither the same vocalic content nor the same approximate length. This prevents relevant comparison between samples and creates biases between them. Finally, this paradigm does not guarantee a minimal length of the samples: on our first attempts to design such a corpus, we have faced patients that answered the quickest possible way to our questions, leading to very short vocal samples, useless to make a corpus.

On the other hand, spontaneous speech benefits from its proximity to ecological conditions and the analysis of the content of speech. Indeed, a recent study in depression detection has linked the augmentation of pronouns and negatively valenced language with the mood of patients affected by bipolar disorders, making it possible to detect through voice (70). Such a system could be developed for sleepiness and excessive sleepiness estimation with the detection of words such as “tired,” “exhausted,” and “sleepy.”

Reading tasks ensure that recordings have the same sizes, the same content and that they are less polluted by emotions. This task seems difficult to implement in an ecological condition, but it allows to study the voice of the subjects in a perfectly controlled environment and with comparable contents, before extending the scope to more “natural” speaking. Moreover, it allows the comparison with a reference medium, to design new biomarkers such as reading errors for example in Martin et al. (15). Finally, on a preliminary study of the MWTc based on spontaneous speech, sleepy patients spoke little if ever: reading texts guarantee the recording of a minimum speech content length.

#### 5.3.2. Performances Depending on the Speech Material on Depression Classification

To our knowledge, no comparative study on the topic of sleepiness has already been made. On the contrary, in the field of depression detection through voice, a previous study studied both reading speech (the Grandfather Passage) and spontaneous speech (patients telling how they feel emotionally and physically) to estimate depression in patients under treatments (71). They reported an accuracy of 64.3% using reading speech against 71.4% for spontaneous speech, seeming to encourage the use of spontaneous speech when designing such corpora. On the contrary, another study (72) based on the reading of the fable “Northwind and the sun,” and a 5–10 min interview for the spontaneous speech, has reported similar performances in the two modalities (83 and 85% for reading and spontaneous speech, respectively), encouraging equally both practices.

Moreover, these two modalities were detected with different vocal features, namely prosody features for spontaneous speech and formant-based features for readings. This is in line with the observation made in Martin et al. (21) on the read parts of the SLC: the features have to be adapted to the type of recorded vocal production. In conclusion, both paradigms have benefits and harms: while reading texts keeps the advantage to guarantee a minimum and comparable vocal production along with the recording sessions, spontaneous speech benefits from its closeness with ecological conditions.

#### 5.3.3. Choice of the Text

This part discusses the choice of such a text. Indeed, to allow speaker state or trait estimation through voice, the chosen texts have to comply with the following constraints.

The chosen texts have to be different for each session of the recordings. Indeed, in the case of the MSLT or the MWT, if the text is the same for all the iterations, there would be both obvious learning and fatigue effects, due to the repeat. However, the texts have to be as similar as possible to keep away a bias due to the type of the text.

First, one of the common points they should have is their length: having different lengths, like it is the case in the MWTc, could lead to bias in terms of fatigue level and time to maintain the needed attention necessary to read. Second, in the MWTc, the content is completely different from one iteration to another, creating biases of emotions that can later pollute the recordings. Indeed, one does not feel the same reading a fable or a vulgarization article: this emotional state, combined with the difference of size, has—partly—prevented us from using the MWTc in sleepiness estimation through voice.

Moreover, the content should not interfere with the sleepiness measure: a stimulating or boring text could change the level of sleepiness of the speaker and could change the result of the KSS, the MWT, or the MSLT sleep latency for example. As a consequence, the chosen texts have to present contents that induce the same emotional state for the reader, both for the vocal production and the medical test.

Besides length and emotional content, other parameters are to be taken into account. For example, the two fables read in the MWTc and the five texts read in the MSLTc are not equivalent as in both cases, some of them have dialogues whereas others do not: since dialogues need a different level of visuo-attentional competencies, the texts have different difficulties, resulting in different readings. To avoid such disparities, we recommend choosing texts without dialogues to ensure that all the patients are on equal terms concerning this point.

Likewise, the MSLT is based on *Le Petit Prince* as it has simple grammar and vocabulary, to prevent the reading level of the speakers from interfering with the detection of sleepiness through the reading: this practice should be generalized to every corpus based on reading texts. To conclude, we do not see interest in proposing a supplementary difficulty to speakers by proposing them texts that are not in their native language: the chosen texts should be in the language spoken by the participant, i.e., the main language of the country in which the study is conducted.

#### 5.3.4. Conclusion and Recommendations on the Speech Task

- **Recording material**
  - Read speech allows to control the content of the recordings, but is away from ecological conditions;
  - Spontaneous speech results in different recording contents but allows analysis of speech.

- **Texts content**
  - Texts should be as similar as possible on emotional and phonetic aspects, with simple grammar and vocabulary.

### 5.4. Length of the Audio Samples

Facing the diversity of length of samples on the four corpora, we investigated this crucial point. Indeed, answering this question allows at the same time to design the vocal task (i.e., length of the text or minimum length of the answer of spontaneous speech), and to choose the length of the chunks when slicing samples to augment data. In the SLEEP corpus, all the samples have a length under 5 s, with a mean of 3.87 s. Indeed, these recordings come from longer audio samples that were sliced into chunks of approximately 4 s. The same practice is usually employed in systems designed for depression (9, 73), in which this sample length has been demonstrated to maximize the accuracy of the employed dataset. Another study on the same task uses chunks of 10 s (74), but the goal behind this choice is not clearly expressed. However, sleepiness detection through voice is a completely different task and sleepiness could manifest differently in voice compared with depression: the question of the length of the samples allowing sleepiness detection still needs to be answered.

#### 5.4.1. Previous Studies

To answer this thorny question, we have already proposed two approaches in the previous study. In Martin et al. (21), after having trained a classifier on the SLC with custom-designed features that achieved performances in the same order as the state of the art, we studied the performances during the inference depending on the length of the samples. The best performances were obtained with samples longer than 8 s. We first proposed this value as the minimum length for the samples. In Martin et al. (14), we sliced the audio samples into increasing-sized chunks and computed the cosine similarity between the features vector of a sample of length n seconds and the same sample of length n-1 s. This led to the conclusion that the minimum length for the features to converge (i.e., the cosine similarity reaches 0.95 and stays above this limit) was 8 s. To do so, we have used both a custom set of features and the openSMILE IS11 features set, which is the reference set of features in the literature (17). The main bias of this technique is that all the features do not have the same scales, and in the end, only the biggest features direct the convergence of the cosine similarity. To overcome this obstacle, we propose in this study a complementary measure, based on statistical tests.

#### 5.4.2. Method

**Figure 8:**
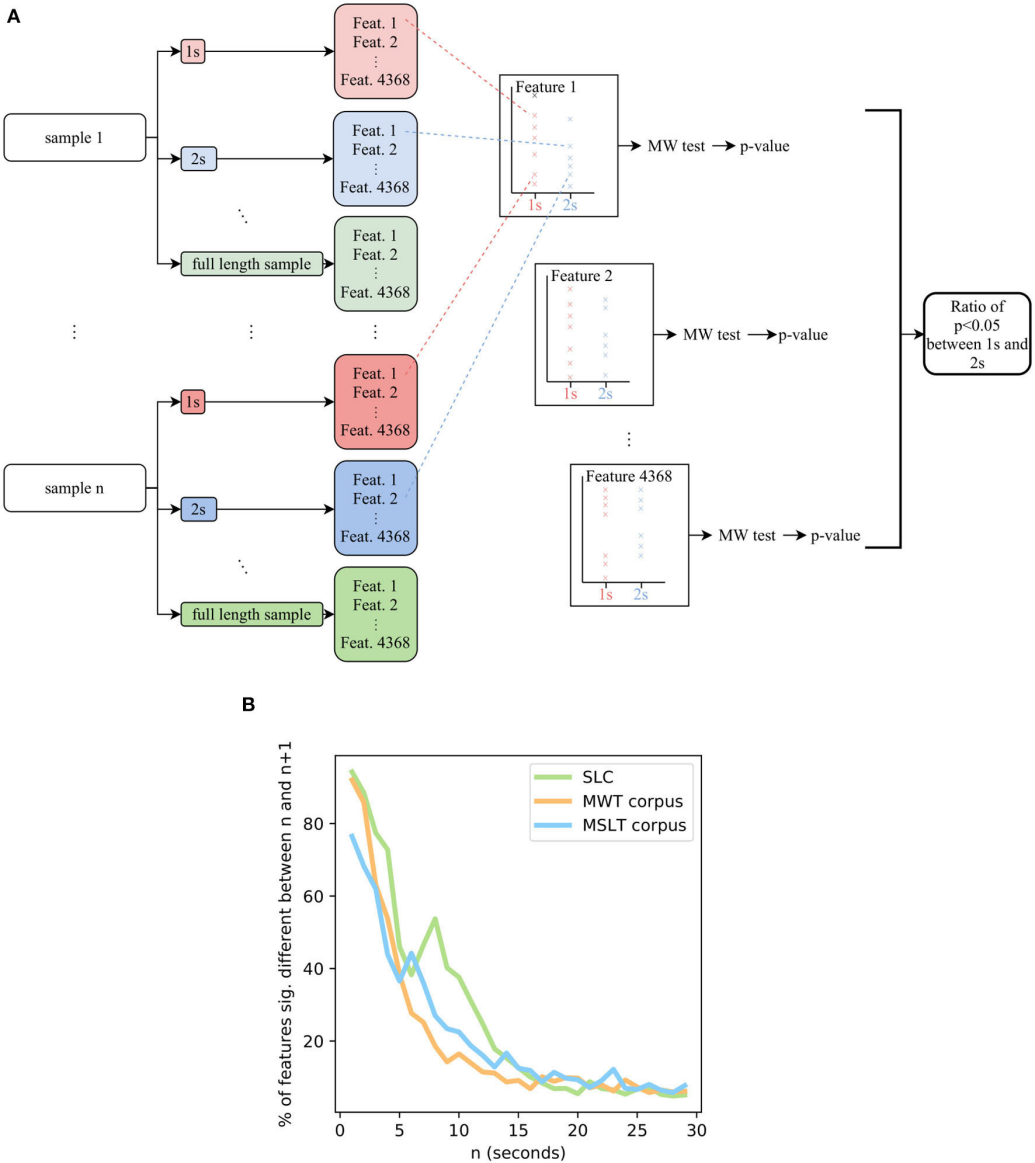
**(A)** Flowchart of the system aiming at estimating the length of minimal audio samples for the computed features to converge. **(B)** The ratio of features having converged depending on the length of the samples, on the SLC, MWTc, and MSLTc. *MW*, Mann-Whitney's U.

Our technique is summarized in Figure 8A. First, all the audio samples are sliced into audio sub-samples of length 1, 2, 3 s, etc. On each of these sub-samples, the state-of-the-art IS11 features are extracted with the openSMILE toolbox (75). Then, for each length *n* s, for each feature *i*, we process a Mann-Whitney test between the features *i* extracted from samples having a length of *n* s and the features *i* extracted from samples having a length of *n* + 1 s. Applying this procedure for all the 4,368 features, we compute the ratio of features for which the distribution for *n* s and *n* + 1 s are significantly different (*p* < 0.05). When it is the case, a supplementary 1-s length to the audio sample results in significant variations of the features, meaning that they did not reach convergence: increasing the length of the sample still brings new information.

#### 5.4.3. Results

Doing so on the four corpora, we obtain the Figure 8B. The SLEEP corpus contains samples lasting less than 5 s, the corresponding graph ends at 5 s with more than 75% of the IS 11 features not having converged. As a consequence, we did not plot it in the graph. Concerning the three other corpora, a length of 20 s leads to ratios less than 15% of features not having converged. According to this metric, the 8 s duration that we previously set up leads to more than 25% of features not having converged yet on the MSLT database and the SLC, and more than 15% for the MWT database: regarding this new metric, an 8 s threshold does not seem enough to guarantee the stationarity of the features. As a consequence, we recommend a minimum length of 20 s.

Taking into account that the IS11 features include complex features comprising double derivatives, these peculiar features need time to be correctly computed and to converge. Although, even if taking into account these features, we encourage machine learning system designers to test if the features fit the sample length of the database they are using. Being aware that the convergence of the features is not directly connected to the sleepiness of the speaker, we assume that regardless of what is to be detected through voice, working with features that would change if the audio sample could have been longer is perilous. Indeed, we have observed the same results for other sets of features available in openSMILE, namely IS09 for emotion recognition, IS10 for depression classification, IS12 for speaker traits estimation, and IS13 ComParE features. As a consequence, independently from the task and the associated features, a minimum length is needed for the features to converge. Moreover, every sample being longer than this minimal length would have the same features, leading to the same interpretation: in case of absence of consensus on this question, we recommend working with samples that are at least 20 s long, to guarantee that the features reached stationarity.

#### 5.4.4. Length of Samples for Depression Detection

Another argument in favor of the use of this threshold is the result obtained in a study dedicated to the length of samples for depression detection through spontaneous speech (76). Studying the influence of the length (number of words) on the performances of two deep learning-based classifiers, they conclude that 1) the minimum length to ensure a generalization of the concept is about 30–50 words (about 20 s in the used corpora), and 2) the accuracy increases with the length of the utterances.

#### 5.4.5. Maximum Length

Nevertheless, we have to warn against too long texts: some patients are easily bored, and too-long text could result in the expression of fatigue or irritations in voice, biasing the measure of sleepiness in it. Moreover, in line with our opinion, the previously cited article (76) shows that accuracy saturates for speeches longer than 120–200 words. As a consequence, we recommend sticking to reasonable lengths: maximal lengths between 1 and 2 min seem reasonable.

#### 5.4.6. Conclusion and Recommendations on the Length of Audio Samples

- **Samples length**
  - A minimum 20 s seem necessary to have the convergence of the features: we recommend not to slice samples under this length;
  - A maximal length of 1–2 min is enough to have sufficient content without inducing fatigue or boredom of the speaker.

### 5.5. Labeling Data

Having chosen the texts and designed the recording sessions, one fundamental question still needs to be answered: when elaborating a database, how to label data? When working with neuro-psychiatric processes, for which concepts are not directly measurable (“sleepiness,” “emotion,” “fatigue,”…) and the paradigms not easy to formulate, this question is not trivial (77). In the specific case of sleepiness, a relevant way to determine the labeling of data is to ask:

1. What is the phenomenon to be measured? Short-term sleepiness? Sleepiness-related disease? Fatigue? Performances at the wheel? All these tasks seem the same at first glance, but they are medically different, and the answer to this question will depend on the way to measure it;
2. What is the targeted population? This question completes the previous one: the medical tests/questionnaires calibrations are sensible to the population they are applied to.

**Figure 9:**
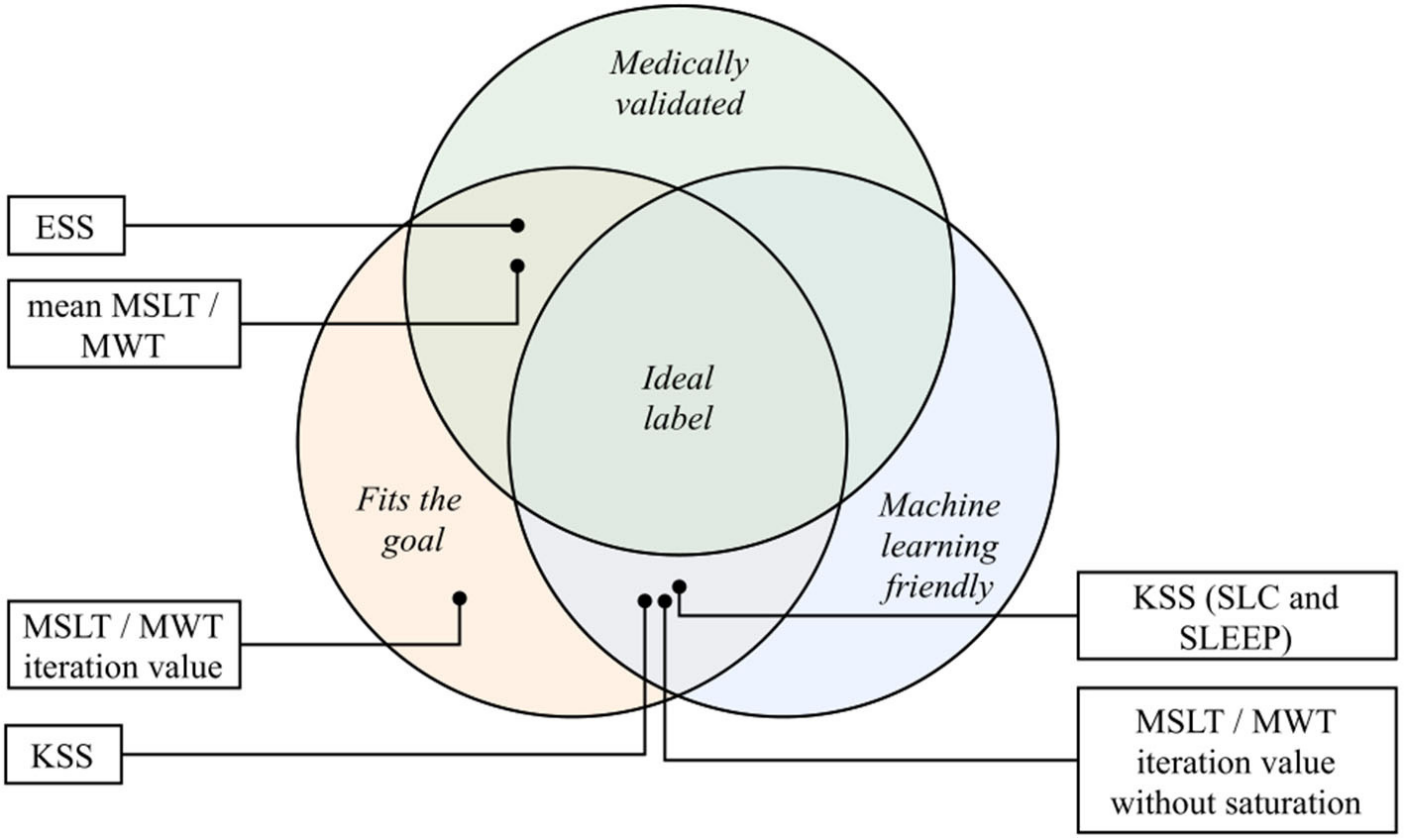
The different sleepiness measures of this study place on the three axes of the ideal label: medical validation, machine learning fitting and goal fitting. KSS, Karolinska Sleepiness Scale; ESS, Epworth Sleepiness Scale; MSLT, Multiple Sleep Latency Test; MWT, Maintenance of Wakefulness Test.

Choosing the right sleepiness measure needs close cooperation between data scientists and physicians (78), to comply with three main constraints: the adequacy between the measure and the objective on the one hand (what is to be measured?), the need for machine learning algorithms to have balanced labels on the other hand (are the labels balanced or unbalanced because of the chosen measure?), and finally, the necessity to keep a medical meaning of the process to finish (need for medical validation). Satisfying the three constraints is an ideal goal but choices have to be made being aware of the compromises they imply. The different sleepiness measures mentioned in this study are represented through these three components in Figure 9.

#### 5.5.1. Sleepiness Measurements Used in the Presented Corpora

The SLC and the SLEEP corpus have been designed to detect short-term sleepiness in healthy subjects. The choice has been made to focus on a mixed sleepiness measurement, scored by both the speaker and two external trained annotators. This score is relatively suited for classification problems and seems relevant for the task it is employed to. The main drawback of this measure relies on its medical validity: the use of KSS as a mixed measure is not, to our knowledge, a medically validated sleepiness measure. The question that still needs to be answered is: what does this value exactly measure?

While the question of the medical meaning of this questionnaire is stills unanswered, achieving great classification performances using it could still lead to new perspectives (the manifestation of sleepiness through voice is not necessarily correlated to reference medical measures of it), but these will have a real meaning only when they will be medically validated and confronted to other measures.

On the MSLTc, the KSS aims at measuring the subjective sleepiness of patients affected by diverse sleep disorders. Fitting the objective of short-term subjective sleepiness detection, this questionnaire suffers from medical validity on pathological populations (79–81), resulting in uncertainty regarding the reliability of the subjective labeling of the speakers.

Compared with the SLC and the SLEEP corpora, the MWTc and MSLTc corpora have been designed with different goals. Indeed, based on validated medical tests, they aim at following patients already being affected by sleep pathologies. The averaged MSLT and MWT sleep latency are both gold standard neurophysiological measures of excessive sleepiness phenomena (51): they both fit the goal of excessive sleepiness measure and are medically validated. Their main drawback is the amount of collected data that makes the elaboration of machine learning systems difficult. Indeed, the collection of these values for one speaker requires multiple recordings throughout the day, and the number of speakers recorded each day depends on the number of recording rooms dedicated to these medical exams. Moreover, as stated in section 4.2.1, the averaged MWT sleep latency suffers from saturation effects, making its machine learning exploitation difficult.

Before averaging them, the independent sleep latency on the MSLT and MWT test could be respective measures of short-term propensity to sleep and altered vigilance. But these measures suffer from two main drawbacks: on the one hand, individual latencies are not medically validated measures of instantaneous objective sleepiness and have not been confronted with other sleepiness-related measures; on the other hand, they suffer from saturation effects (as shown in section 4.2.1). As a consequence, the attempt to use them as a measure of objective short-term sleepiness seems compromised. Collected on the MSLT and MWT corpora, the ESS aims at measuring subjectively pathological propensity to sleep. Medically validated (41) and machine-learning-friendly (refer to distribution on Supplementary Materials), this questionnaire seems to fit the goal of subjectively pathological propensity to sleep label. However, its main drawback is its usage according to the population that it is administered to, leading to different cut-off values.

#### 5.5.2. Other Sleepiness Measurements

The sleepiness measurements do not stop at the six ones studied in this article. Figure 1 completes the list but the tools designed to measure sleepiness are numerous and as diversified as its nature: questionnaires, EEG-based measures, eye-activity-related metrics, behavioral features, … This diversity emphasizes the need for a close collaboration between clinical practitioners and data scientists, to find the labels that exactly fit the requirements of both parties.

For reviews on this topic, one may check the following articles: (30, 31, 82–86).

#### 5.5.3. Binary Classification vs. Regression: Mimic the Medical Practitioners

A relevant way for machine learning engineers to decide to binarize the problem or to keep it as a regression problem could be to mimic the medical practices. Indeed, when some measures are acknowledged as categorical questionnaires, the binarization of the problem using a threshold seems relevant. On the contrary, when clinicians use these scores in a continuous manner, the binarization of the problem is meaningless, even if it eases the machine learning classification. In all the cases, we recommend corpus designer to provide raw measurement in corpora, to allow machine learning engineers and physicians to test different settings.

#### 5.5.4. Metadata

As mentioned in Qian et al. (87), to fully understand the concepts brought into play when working on these corpora, the best practice is to collect numerous data about the speakers and the recording conditions. This allows unraveling biases that are not necessarily identified and to ensure that, when elaborating classifiers, the samples are classified by what they are meant to, and not by a related bias (88, 89). Moreover, these measures allow studying the robustness of systems with respect to other factors (sex, age, demographic data, comorbidity, …). For example, a recent study (90) has re-examined a system aiming at detecting depression through voice, including demographic data and emotions labeling. This has led to the conclusion that the studied system is independent of both demographic data and the emotional state of the speaker: this conclusion could not have been made possible without such information in their corpus. As a conclusion, we encourage to label voice recordings with a maximum of relevant information (physical information about the speaker, comorbidities, demographic data, etc …), to allow at the same time the evaluation of the robustness of elaborated systems to the phenomena measured by these data, but also to correct and study precisely the label used in the database, and to give insights on its manifestation through voice.

#### 5.5.5. Conclusion and Recommendations on Data Labeling

From the discussion of this section, we have identified the following points of interest:

- **Choosing the relevant label**
  - We encourage to emphasize collaborations between the different fields needed to relevantly label data (mainly data scientists and clinical practitioners), avoiding mislabeling of the task and guaranteeing the quality of the labeling (78).

- **Providing raw measurement in corpora**
- **Collect the most possible metadata**
  - As it has been proven useful, we encourage to maximize the relevant collected metadata about the speakers;
  - In the same vein, systematically reporting the population and the experimental conditions in which they have been recorded when communicating about a corpus or a machine learning system allows relevant comparisons and identification of the phenomena.

## Data Availability Statement

The data analyzed in this study is subject to the following licenses/restrictions: The data that support the findings of this study are available from the corresponding author upon reasonable requests. Requests to access these datasets should be directed to Jean-Luc Rouas, rouas@labri.fr.

## Ethics Statement

Ethical approval for this study and written informed consent from the participants of the study were not required in accordance with local legislation and national guidelines.

## Author Contributions

VM and J-LR wrote the manuscript. VM performed the statistics on the corpora. PP and J-AM-F guided the medical parts of the discussion and task statement, whereas JK guided the description of the SLC and the SLEEP corpora. PP is the principal investigator of the Multiple Sleep Latency Test (MSLT) and Maintenance of Wakefulness Test (MWT) corpora studies. JK is the principal supervisor of the Sleepy Language Corpus (SLC) and SLEEP corpora studies. All authors contributed to the article and approved the submitted version.

## Funding

This study is carried out in the framework of the IS Obstructive Sleep Apnea (OSA) project funded by the French Region Nouvelle Aquitaine and by the SOMVOICE project sponsored by the Labex BRAIN (ANR-10-LABX-43). This study was partially supported by the Horizon H2020 My Active and Healthy Ageing, (No. 689592) and German Federal Ministry of Education and Research (BMBF) under grant agreement 01–S15024 (VIVID–IKT2020/2015-2018).

## Conflict of Interest

The authors declare that the research was conducted in the absence of any commercial or financial relationships that could be construed as a potential conflict of interest.

## Publisher's Note

All claims expressed in this article are solely those of the authors and do not necessarily represent those of their affiliated organizations, or those of the publisher, the editors and the reviewers. Any product that may be evaluated in this article, or claim that may be made by its manufacturer, is not guaranteed or endorsed by the publisher.
