# Supplementary material for: How to Design a Relevant Corpus for Sleepiness Detection Through Voice?
Source: Front Digit Health. 2021 Sep 22;3:686068. doi: 10.3389/fdgth.2021.686068 (PMC8521834; doi:10.3389/fdgth.2021.686068)
Supplement: Supplementary file 1 [file Data_Sheet_1.PDF]

Supplementary Material

1 SUPPLEMENTARY DATA

1.1 Figures

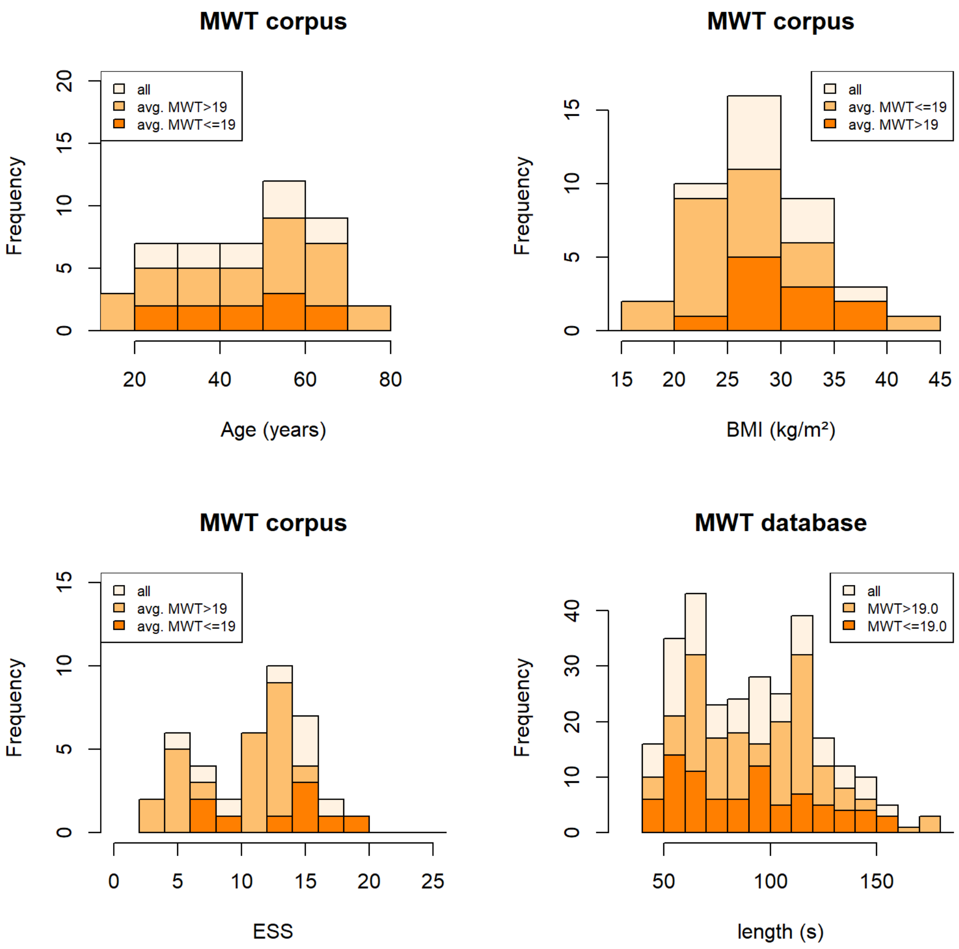

Figure S1. Histograms of the supplementary data collected in the MWT corpus, **(A)** Age (years) **(B)** Body Mass Index (kg/m^2) **(C)** Epworth Sleepiness Score (D) Length of the samples

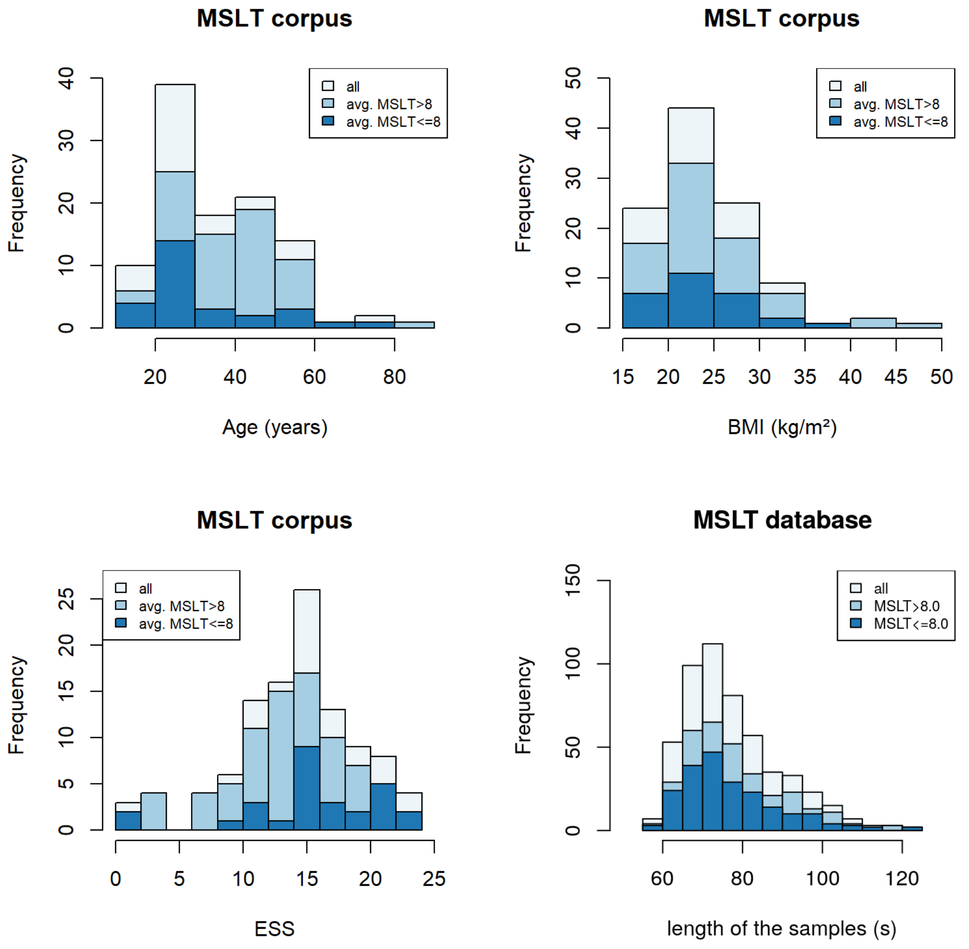

Figure S2. Histograms of the data collected in the MSLT corpus, **(A)** Age (years) **(B)** Body Mass Index (kg/m^2) **(C)** Epworth Sleepiness Score (D) Length of the samples
